# Robust and intelligent control strategies for a 3-DOF robotic arm: a comparative study

**DOI:** 10.1038/s41598-026-53593-2

**Published:** 2026-05-24

**Authors:** Eman E. Esmail, Mohamed Fawzy El-Khatib, M. A. Agwa

**Affiliations:** 1 Mechatronics and Robotics Engineering Department, Faculty of Engineering, Egyptian Russian University, Cairo 11829, Egypt; 2 Mechanical Design and Production Engineering Department, Faculty of Engineering, Zagazig University, Zagazig 44519, Egypt

**Keywords:** Robotic manipulator, Nonlinear control, Sliding mode control, Fuzzy logic control, PID control, Trajectory tracking, Engineering, Mathematics and computing

## Abstract

This paper presents a comparative study of PID, Fuzzy Logic Control (FLC), and Sliding Mode Control (SMC) strategies for trajectory tracking of a nonlinear 3-DOF robotic manipulator. A complete dynamic model is derived using the Euler–Lagrange formulation, incorporating inertia coupling, Coriolis and centrifugal effects, gravitational forces, and joint friction. The developed model is validated against published results, demonstrating close agreement in amplitude and phase characteristics under sinusoidal joint trajectories. The three controllers are implemented under identical conditions and evaluated in both joint space and Cartesian space using step inputs, infinity trajectories, and circular paths. Performance is quantitatively assessed using RMSE, ITAE, and IAE metrics. The results indicate that SMC achieves the highest tracking accuracy, reducing average RMSE by approximately 80% compared to PID, while FLC achieves nearly 50% improvement. In Cartesian tracking, SMC maintains peak position errors below 0.005 m, significantly outperforming PID, which exhibits deviations up to 0.11 m under dynamic motion. Statistical analysis further confirms improved robustness under the considered simulation scenarios and the consistency of SMC across all joints. The findings demonstrate that robust nonlinear control significantly enhances convergence speed, tracking precision, and disturbance rejection capability in planar 3-DOF manipulators. The validated modeling framework and systematic benchmarking provide practical guidance for selecting appropriate control strategies in industrial robotic applications.

## Introduction

Robotic manipulators with multiple degrees of freedom are essential components of contemporary automation systems, enabling accurate motion execution in industrial manufacturing, medical robotics, and autonomous manipulation tasks^1–3^. Their growing deployment in unstructured and dynamically changing environments has intensified the demand for control strategies capable of ensuring high tracking accuracy, robustness to uncertainties, and reliable real-time performance^4^. In this context, the three-degree-of-freedom (3-DOF) robotic arm serves as a representative planar manipulator that captures the fundamental challenges of nonlinear motion control while maintaining analytical and computational tractability^1,2^.

The dynamic behavior of a 3-DOF robotic arm is inherently nonlinear, coupled, and configuration dependent, governed by time-varying inertia matrices, Coriolis and centrifugal forces, and gravity-induced torques^1,3,5^. These characteristics are further complicated by practical factors such as joint friction, actuator limitations, payload variations, and external disturbances^6^. As a result, the design of high-performance controllers for such systems remains an open research problem, particularly when robustness and implementation simplicity must be balanced^4,7^.

Among the wide spectrum of control methodologies proposed for robotic manipulators, Proportional–Integral–Derivative (PID) control continues to dominate industrial practice due to its straightforward structure and ease of deployment^8^. However, fixed-gain PID controllers are fundamentally limited when applied to nonlinear robotic systems operating over wide workspaces. Variations in system dynamics across different configurations can lead to performance degradation, oscillations, or slow transient responses, even when extensive gain tuning is employed^5,8^.

To mitigate the limitations of classical control, Fuzzy Logic Control (FLC) has been extensively explored as an intelligent, model-free alternative^9,10^. By exploiting linguistic rules and approximate reasoning, fuzzy controllers can effectively handle nonlinearities and parameter uncertainties without explicit reliance on an accurate mathematical model^9^. This flexibility has made FLC attractive for robotic applications involving friction effects and unmodeled dynamics. Nevertheless, the design of fuzzy controllers often depends on heuristic tuning of membership functions and rule bases, and rigorous stability guarantees are rarely established, raising concerns regarding predictability and certification in demanding robotic tasks^11^.

In contrast, Sliding Mode Control (SMC) offers a theoretically grounded framework for robust nonlinear control, providing strong invariance properties against matched uncertainties and disturbances^12–14^. By driving the system trajectories onto a predefined sliding manifold, SMC ensures finite-time convergence and robustness^12^. Despite these advantages, the discontinuous control action inherent to conventional SMC gives rise to the well-known chattering phenomenon, which may excite unmodeled dynamics, increase actuator stress, and degrade long-term system reliability^13,14^. Although continuous and higher-order sliding mode schemes have been proposed to alleviate chattering, they often introduce additional complexity and tuning challenges that limit their practical adoption^15^. Recent advancements in sliding mode control have introduced several enhanced variants, including terminal sliding mode control, fixed-time and finite-time SMC, and disturbance observer-based approaches, which improve convergence speed, robustness, and chattering mitigation. For instance, disturbance observer-based SMC has been shown to effectively compensate for modeling uncertainties and external disturbances, while fixed-time SMC guarantees convergence within a predefined time independent of initial conditions. Despite these advantages, such methods often involve increased design complexity and computational burden. Therefore, this study adopts a classical sliding mode control formulation to provide a clear and consistent baseline for comparative evaluation^16–20^.

While PID, fuzzy logic, and sliding mode control have each been widely investigated, systematic and high-fidelity comparative studies for planar 3-DOF manipulators remain limited. Many existing works rely on simplified planar models, neglect gravity-induced coupling, or evaluate performance using isolated metrics such as tracking error alone^16–20^. Moreover, the literature lacks clear guidance on the performance–complexity trade-off, which is crucial for selecting an appropriate control strategy in real-world robotic applications where computational resources, robustness, and ease of implementation must be jointly considered^21–25^.

Recent advances in nonlinear control have introduced several enhanced strategies for robotic manipulators to address system uncertainties and improve tracking performance. Backstepping control has been widely adopted due to its recursive design methodology and strong stability guarantees for nonlinear systems^26–28^. In addition, adaptive and intelligent control approaches, particularly adaptive neural network-based control, have been developed to approximate unknown nonlinear dynamics and enhance robustness^29^. Recent works have further extended these approaches by integrating fixed-time convergence properties and prescribed performance constraints, enabling guaranteed tracking accuracy within a predefined time independent of initial conditions^20^. Moreover, disturbance observer-based control strategies have been combined with fixed-time control to estimate and compensate for lumped uncertainties and external disturbances in real time^20^. Despite their effectiveness, these advanced methods often involve increased computational complexity and require careful parameter tuning, which may limit their practical implementation in real-time applications. Therefore, this study focuses on a structured comparative evaluation of PID, FLC, and SMC as representative control strategies with different levels of complexity and robustness.

In this context, several studies have investigated the comparative performance of control strategies for robotic manipulators, particularly in trajectory tracking applications^21,23,30^. These works typically evaluate different controllers based on tracking accuracy, robustness, or computational complexity under specific operating conditions. However, many existing comparative studies are limited to simplified models, specific control structures, or a restricted set of performance metrics. In addition, the lack of unified evaluation frameworks and consistent testing conditions makes it difficult to draw general conclusions regarding controller performance. This motivates the need for a systematic and fair comparative analysis under identical dynamic conditions, which is addressed in this work.

This paper aims to address these limitations through a systematic comparative analysis by presenting a unified and rigorous comparative investigation of PID, Fuzzy Logic, and Sliding Mode Control applied to a nonlinear 3-DOF robotic arm. The main contributions are summarized as follows:

1. A unified and fair comparative framework for evaluating classical (PID), intelligent (FLC), and robust nonlinear (SMC) controllers under identical dynamic conditions, including both joint-space and Cartesian-space tracking tasks.
2. A high-fidelity nonlinear simulation platform incorporating full dynamic coupling and validated against published results, enabling consistent benchmarking across multiple trajectory profiles.
3. A comprehensive multi-metric evaluation combining transient response, RMSE, ITAE, and statistical error analysis to provide deeper insight into performance–robustness trade-offs in robotic manipulators.

The remainder of this paper is organized as follows. Section 2 presents the nonlinear dynamic modeling of the 3-DOF robotic manipulator using the Euler–Lagrange formulation. Section 3 describes the design and implementation of the PID, Fuzzy Logic, and Sliding Mode control strategies. Section 4 provides the validation of the developed dynamic model against published results to ensure modeling accuracy. Section 5 presents the simulation results and comparative performance analysis in both joint space and Cartesian space. Finally, Sect. 6 concludes the paper and outlines future research directions.

## Dynamic modeling of the 3-DOF robotic manipulator

This section presents the complete nonlinear dynamic modeling of the three-degree-of-freedom (3-DOF) robotic manipulator using the Euler–Lagrange formulation. The objective is to obtain an accurate and control-oriented mathematical representation of the system dynamics that can be directly implemented in MATLAB/Simulink for controller design and comparative performance evaluation. The Euler–Lagrange approach provides a systematic method for deriving the equations of motion of multi-link robotic systems by explicitly accounting for the kinetic and potential energies of each rigid link^1–4^. This formulation naturally captures the strong nonlinearities and coupling effects inherent in articulated manipulators, which are critical for high-fidelity simulation and robust control synthesis^1,2^.

### System description and modeling assumptions

**Fig. 1:**
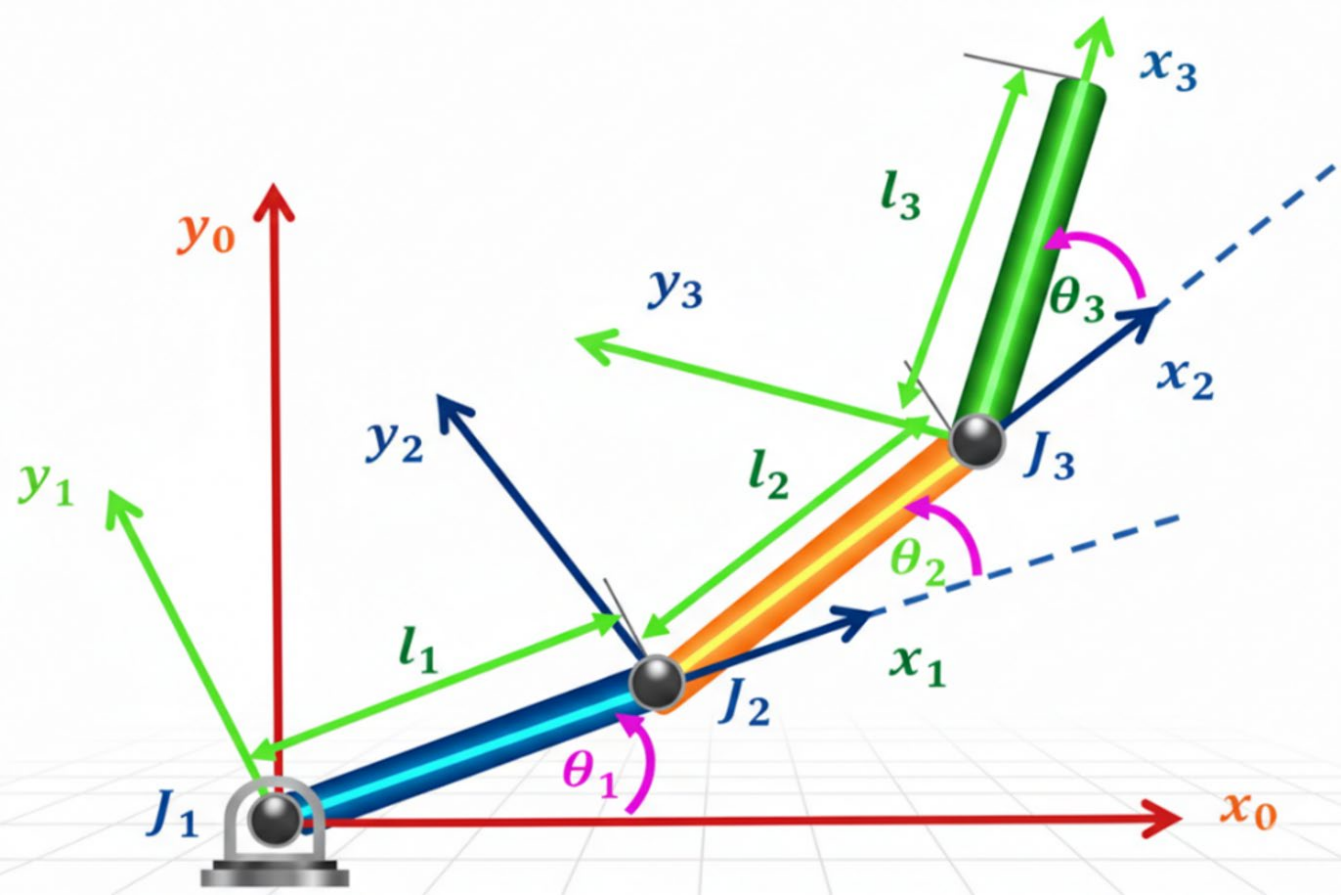
Schematic representation of the 3-DOF robotic manipulator.

The robotic system under consideration is a planar serial manipulator composed of three rigid links connected by revolute joints, providing three rotational degrees of freedom. The first link is attached to a fixed base through an ideal pin joint, while the second and third links are connected via revolute joints. For modeling simplicity, ideal joint behavior is initially assumed; however, friction effects are incorporated in the dynamic model to ensure realistic system representation. The joint variables are denoted by $\:{\theta\:}_{1}$, $\:{\theta\:}_{2}$, and $\:{\theta\:}_{3}$, measured with respect to their preceding links. Each link is assumed to be rigid and homogeneous, with its mass uniformly distributed along its length and its center of gravity located at the midpoint of the link. The moments of inertia are defined about the center of gravity of each link. Gravitational forces act on all links, and control torques are applied at the joints^4,5^. The physical parameters of the manipulator are listed in Table 1, while the geometric configuration and coordinate frames are illustrated in Fig. 1.

**Table 1:** Physical and inertial parameters of the 3-link robotic manipulator.

Parameter	Symbol	Unit	Link 1	Link 2	Link 3
Mass	mi	kg	1.0	1.0	1.0
Link Length	Li	m	1.0	1.0	0.5
Moment of Inertia	Ii	kg·m²	0.10	0.10	0.10

For clarity and consistency, the principal symbols used in the dynamic modeling and control design are summarized in Table 2.

**Table 2:** Notation used in the dynamic model.

Symbol	Description	Unit
$\:\boldsymbol{\theta\:}=[{\theta\:}_{1}{\hspace{0.25em}\hspace{0.05em}}{\theta\:}_{2}{\hspace{0.25em}\hspace{0.05em}}{\theta\:}_{3}{]}^{T}$	Joint angle vector	rad
$\:\boldsymbol{\theta\:},\dot{\boldsymbol{\theta\:}}$	Joint velocity and acceleration	rad/s, rad/s²
$\:\mathbf{M}\left(\boldsymbol{\theta\:}\right)$	Inertia matrix	kg·m²
$\:\mathbf{C}(\boldsymbol{\theta\:},\dot{\boldsymbol{\theta\:}})$	Coriolis and centrifugal matrix	—
$\:\mathbf{G}\left(\boldsymbol{\theta\:}\right)$	Gravity torque vector	N·m
$\:\boldsymbol{F}\left(\dot{\boldsymbol{\theta\:}}\right)$	Friction vector	N·m
$\:\boldsymbol{\tau\:}$	Control input torque	N·m
$\:e$	Tracking error	rad
$\:\dot{e}$	Velocity error	rad/s
**S**	Sliding surface	—
$\:\boldsymbol{\Lambda\:}$	Sliding surface gain matrix	—
$\:K$	SMC switching gain matrix	—
$\:\phi\:$	Boundary layer thickness	—

These symbols are used consistently throughout the manuscript unless otherwise stated.

### Euler–lagrange dynamic formulation

Let the generalized coordinate vector be defined as $\:\boldsymbol{\theta\:}=[{\theta\:}_{1}{\hspace{0.25em}\hspace{0.05em}}{\theta\:}_{2}{\hspace{0.25em}\hspace{0.05em}}{\theta\:}_{3}{]}^{T}$. Using the Euler–Lagrange formulation, the nonlinear equations of motion of the robotic manipulator can be expressed in the standard robotic form as

1 $$\:\mathbf{M}\left(\boldsymbol{\theta\:}\right){\hspace{0.17em}}\ddot{\boldsymbol{\theta\:}}+\boldsymbol{C}\left(\boldsymbol{\theta\:},\dot{\boldsymbol{\theta\:}}\right)\dot{\boldsymbol{\theta\:}}+\mathbf{G}\left(\boldsymbol{\theta\:}\right)\:+\boldsymbol{F}\left(\dot{\boldsymbol{\theta\:}}\right)=\boldsymbol{\tau\:}$$

where $\:\mathbf{M}\left(\boldsymbol{\theta\:}\right)$ is the inertia matrix, $\:\mathbf{C}(\boldsymbol{\theta\:},\dot{\boldsymbol{\theta\:}})$ represents the combined centrifugal and Coriolis effects, $\:\mathbf{G}\left(\boldsymbol{\theta\:}\right)$ is the gravitational torque vector, $\:\boldsymbol{F}\left(\dot{\boldsymbol{\theta\:}}\right)$ denotes friction forces, and $\:\boldsymbol{\tau\:}=[{\tau\:}_{1}{\hspace{0.25em}\hspace{0.05em}}{\tau\:}_{2}{\hspace{0.25em}\hspace{0.05em}}{\tau\:}_{3}{]}^{T}$denotes the vector of joint control torques.

### Inertia matrix elements

The inertia matrix $\:\mathbf{M}\left(\boldsymbol{\theta\:}\right)\in\:{\mathbb{R}}^{3\times\:3}$ is symmetric and positive definite. Its elements are given explicitly as follows:

2 $$\:\begin{array}{cc}{M}_{11}&\:={l}_{1}^{2}(0.25{m}_{1}+{m}_{2}+{m}_{3})+{l}_{2}^{2}(0.25{m}_{2}+{m}_{3})+0.25{l}_{3}^{2}{m}_{3}\\\:&\:+{l}_{1}{l}_{2}\mathrm{c}\mathrm{o}\mathrm{s}\left({\theta\:}_{2}\right)({m}_{2}+2{m}_{3})+{l}_{2}{l}_{3}{m}_{3}\mathrm{c}\mathrm{o}\mathrm{s}\left({\theta\:}_{3}\right)\\\:&\:+{l}_{1}{l}_{3}{m}_{3}\mathrm{c}\mathrm{o}\mathrm{s}({\theta\:}_{2}+{\theta\:}_{3})+{I}_{1}+{I}_{2}+{I}_{3}\end{array}$$

3 $$\:\begin{array}{cc}{M}_{12}&\:={l}_{2}^{2}(0.25{m}_{2}+{m}_{3})+0.25{l}_{3}^{2}{m}_{3}+{l}_{1}{l}_{2}\mathrm{c}\mathrm{o}\mathrm{s}\left({\theta\:}_{2}\right)({m}_{3}+0.5{m}_{2})\\\:&\:+{l}_{2}{l}_{3}{m}_{3}\mathrm{c}\mathrm{o}\mathrm{s}\left({\theta\:}_{3}\right)+0.5{l}_{1}{l}_{3}{m}_{3}\mathrm{c}\mathrm{o}\mathrm{s}({\theta\:}_{2}+{\theta\:}_{3})+{I}_{2}+{I}_{3}\end{array}$$

4 $$\:{M}_{13}=0.25{l}_{3}^{2}{m}_{3}+0.5{l}_{2}{l}_{3}{m}_{3}\mathrm{cos}\left({\theta\:}_{3}\right)+0.5{l}_{1}{l}_{3}{m}_{3}\mathrm{cos}\left({\theta\:}_{2}+{\theta\:}_{3}\right)+{I}_{3}$$

5 $$\:{M}_{21}={M}_{12}$$

6 $$\:{M}_{22}={l}_{2}^{2}(0.25{m}_{2}+{m}_{3})+0.25{l}_{3}^{2}{m}_{3}+{l}_{2}{l}_{3}{m}_{3}\mathrm{c}\mathrm{o}\mathrm{s}\left({\theta\:}_{3}\right)+{I}_{2}+{I}_{3}$$

7 $$\:{M}_{23}=0.25{l}_{3}^{2}{m}_{3}+0.5{l}_{2}{l}_{3}{m}_{3}\mathrm{c}\mathrm{o}\mathrm{s}\left({\theta\:}_{3}\right)+{I}_{3}$$

8 $$\:{M}_{31}={M}_{13};\:{M}_{32}={M}_{23}$$

9 $$\:{M}_{33}=0.25{l}_{3}^{2}{m}_{3}+{I}_{3}$$

These expressions highlight the configuration-dependent nature of the inertia matrix and the strong coupling between joints.

### Coriolis and centrifugal forces

The nonlinear vector $\:\mathbf{C}(\boldsymbol{\theta\:},\dot{\boldsymbol{\theta\:}})$ accounts for centrifugal and Coriolis effects and is defined by the following components:

10 $$\:\begin{array}{cc}{C}_{1}(\boldsymbol{\theta\:},\dot{\boldsymbol{\theta\:}})&\:=-{\dot{\theta\:}}_{2}^{2}\left(0.5{l}_{1}{l}_{2}{m}_{2}\mathrm{sin}\left({\theta\:}_{2}\right)+{l}_{1}{l}_{2}{m}_{3}\mathrm{sin}\left({\theta\:}_{2}\right)+0.5{l}_{1}{l}_{3}{m}_{3}\mathrm{s}\mathrm{i}\mathrm{n}({\theta\:}_{2}+{\theta\:}_{3})\right)\\\:&\:-{\dot{\theta\:}}_{3}^{2}\left(0.5{l}_{1}{l}_{3}{m}_{3}\mathrm{s}\mathrm{i}\mathrm{n}({\theta\:}_{2}+{\theta\:}_{3})\right)\\\:&\:-{\dot{\theta\:}}_{1}{\dot{\theta\:}}_{2}\left({l}_{1}{l}_{2}{m}_{2}\mathrm{sin}\left({\theta\:}_{2}\right)+2{l}_{1}{l}_{2}{m}_{3}\mathrm{sin}\left({\theta\:}_{2}\right)+{l}_{1}{l}_{3}{m}_{3}\:\mathrm{s}\mathrm{i}\mathrm{n}({\theta\:}_{2}+{\theta\:}_{3})\right)\\\:&\:-{\dot{\theta\:}}_{1}{\dot{\theta\:}}_{3}\left({l}_{1}{l}_{3}{m}_{3}\mathrm{sin}\left({\theta\:}_{2}+{\theta\:}_{3}\right)+{l}_{2}{l}_{3}{m}_{3}\:\mathrm{sin}\left({\theta\:}_{3}\right)\:\right)\\\:&\:-{\dot{\theta\:}}_{2}{\dot{\theta\:}}_{3}\:({l}_{1}{l}_{3}{m}_{3}\mathrm{sin}\left({\theta\:}_{2}+{\theta\:}_{3}\right)+{l}_{2}{l}_{3}{m}_{3\:}\mathrm{sin}\left({\theta\:}_{3}\right)\end{array}$$

11 $$\:\begin{array}{cc}{C}_{2}(\boldsymbol{\theta\:},\dot{\boldsymbol{\theta\:}})&\:={\dot{\theta\:}}_{1}^{2}\left(0.5{l}_{1}{l}_{2}{m}_{2}\mathrm{sin}\left({\theta\:}_{2}\right)+{l}_{1}{l}_{2}{m}_{3}\mathrm{sin}\left({\theta\:}_{2}\right)+0.5{l}_{1}{l}_{3}{m}_{3}\:\mathrm{s}\mathrm{i}\mathrm{n}({\theta\:}_{2}+{\theta\:}_{3})\right)\\\:&\:-0.5{\dot{\theta\:}}_{3}^{2}{l}_{2}{l}_{3}{m}_{3}\mathrm{s}\mathrm{i}\mathrm{n}\left({\theta\:}_{3}\right)-{\dot{\theta\:}}_{1}{\dot{\theta\:}}_{3}{l}_{2}{l}_{3}{m}_{3}\mathrm{s}\mathrm{i}\mathrm{n}\left({\theta\:}_{3}\right)\end{array}$$

12 $$\:\begin{array}{cc}{C}_{3}(\boldsymbol{\theta\:},\dot{\boldsymbol{\theta\:}})&\:={\dot{\theta\:}}_{1}^{2}\left(0.5{l}_{2}{l}_{3}{m}_{3}\mathrm{s}\mathrm{i}\mathrm{n}\left({\theta\:}_{3}\right)+0.5{l}_{1}{l}_{3}{m}_{3}\mathrm{s}\mathrm{i}\mathrm{n}({\theta\:}_{2}+{\theta\:}_{3})\right)\\\:&\:+0.5{\dot{\theta\:}}_{2}^{2}{l}_{2}{l}_{3}{m}_{3}\mathrm{s}\mathrm{i}\mathrm{n}\left({\theta\:}_{3}\right)+{\dot{\theta\:}}_{1}{\dot{\theta\:}}_{2}{l}_{2}{l}_{3}{m}_{3}\mathrm{s}\mathrm{i}\mathrm{n}\left({\theta\:}_{3}\right)\end{array}$$

These nonlinear terms significantly affect transient behavior and must be explicitly considered in controller design.

### Gravitational torque vector

The gravitational torque vector $\:\mathbf{G}\left(\boldsymbol{\theta\:}\right)$ is given by:

13 $$\:\begin{array}{cc}{g}_{1}\left(\boldsymbol{\theta\:}\right)&\:={m}_{3}g\left({l}_{1}\mathrm{c}\mathrm{o}\mathrm{s}\left({\theta\:}_{1}\right)+0.5{l}_{3}\mathrm{c}\mathrm{o}\mathrm{s}({\theta\:}_{1}+{\theta\:}_{2}+{\theta\:}_{3})+{l}_{2}\mathrm{c}\mathrm{o}\mathrm{s}({\theta\:}_{1}+{\theta\:}_{2})\right)\\\:&\:+{m}_{2}g\left({l}_{1}\mathrm{c}\mathrm{o}\mathrm{s}\left({\theta\:}_{1}\right)+0.5{l}_{2}\mathrm{c}\mathrm{o}\mathrm{s}({\theta\:}_{1}+{\theta\:}_{2})\right)+0.5{m}_{1}g{l}_{1}\mathrm{c}\mathrm{o}\mathrm{s}\left({\theta\:}_{1}\right)\end{array}$$

14 $$\:\begin{array}{cc}{g}_{2}\left(\boldsymbol{\theta\:}\right)&\:={m}_{3}g\left(0.5{l}_{3}\mathrm{cos}\left({\theta\:}_{1}+{\theta\:}_{2}+{\theta\:}_{3}\right)+{l}_{2}\mathrm{cos}\left({\theta\:}_{1}+{\theta\:}_{2}\right)\right)\\\:&\:+0.5{m}_{2}g{l}_{2}\mathrm{cos}\left({\theta\:}_{1}+{\theta\:}_{2}\right)\end{array}$$

15 $$\:{g}_{3}\left(\boldsymbol{\theta\:}\right)=0.5{m}_{3}g{l}_{3}\mathrm{c}\mathrm{o}\mathrm{s}({\theta\:}_{1}+{\theta\:}_{2}+{\theta\:}_{3})$$

where $\:g$ denotes the gravitational acceleration.

### Friction forces

The Friction Forces $\:\boldsymbol{F}\left(\dot{\boldsymbol{\theta\:}}\right)$ is given by:

16 $$\:{f}_{1}\left(\dot{\boldsymbol{\theta\:}}\right)={b}_{1}{\dot{\theta\:}}_{1}+\:{k}_{f1}\:\mathrm{t}\mathrm{a}\mathrm{n}\mathrm{h}\left(\alpha\:{\dot{\theta\:}}_{1}\right)$$

17 $$\:{f}_{2}\left(\dot{\boldsymbol{\theta\:}}\right)={b}_{2}{\dot{\theta\:}}_{2}+\:{k}_{f2}\:\mathrm{t}\mathrm{a}\mathrm{n}\mathrm{h}\left(\alpha\:{\dot{\theta\:}}_{2}\right)$$

18 $$\:{f}_{3}\left(\dot{\boldsymbol{\theta\:}}\right)={b}_{3}{\dot{\theta\:}}_{3}+\:{k}_{f3}\:\mathrm{t}\mathrm{a}\mathrm{n}\mathrm{h}\left(\alpha\:{\dot{\theta\:}}_{3}\right)$$

where $\:{b}_{i}$represents the viscous friction coefficient for the $\:i$-th joint, $\:{k}_{fi}$denotes the Coulomb friction coefficient, and $\:\alpha\:$is a smoothing factor used to ensure a continuous and stable transition around zero velocity.

### Simulink-oriented representation

Equations (1)–(18) form a complete nonlinear dynamic representation of the 3-DOF robotic manipulator and are directly implemented in MATLAB/Simulink. The explicit separation of inertia, Coriolis/centrifugal, and gravity terms enables modular block implementation and facilitates integration with classical and nonlinear control strategies.

This formulation provides a reliable and computationally efficient platform for the comparative evaluation of PID, Fuzzy Logic, and Sliding Mode Controllers, which is addressed in the subsequent sections.

## Control system design

This section describes the control strategies developed to regulate the motion of the three-degree-of-freedom (3-DOF) robotic arm. The main objective is to achieve accurate joint-space trajectory tracking despite the presence of nonlinear dynamics, strong coupling between joints, gravitational effects, and external disturbances. Based on the nonlinear dynamic model derived in Sect. 2, three control approaches are considered: PID control, fuzzy logic control, and sliding mode control. These methods represent classical, intelligent, and robust nonlinear control philosophies, respectively, and are selected to provide a clear and meaningful performance comparison under identical operating conditions.

Let the desired joint trajectory be defined as

19 $$\:{\boldsymbol{\theta\:}}_{\boldsymbol{d}}\left(\boldsymbol{t}\right)={\left[{\theta\:}_{1d}\left(t\right)\:\:{\theta\:}_{2d}\left(t\right)\:\:{\theta\:}_{3d}\left(t\right)\right]}^{T}$$

and the tracking error vector as

20 $$\:\boldsymbol{e}\left(\boldsymbol{t}\right)=\boldsymbol{\theta\:}\boldsymbol{d}\boldsymbol{}\left(\boldsymbol{t}\right)-\boldsymbol{\theta\:}\left(\boldsymbol{t}\right)$$

The desired velocity trajectory is obtained as the time derivative of the desired position trajectory, i.e., $\:{\dot{\theta\:}}_{d}\left(t\right)=\frac{d}{dt}{\theta\:}_{d}\left(t\right)$. Accordingly, both position and velocity tracking errors are considered in the control design and performance evaluation.

The control objective is to design the joint torque input $\:\boldsymbol{\tau\:}\left(\boldsymbol{t}\right)$ such that the tracking errors converge to zero while maintaining bounded control effort and stable closed-loop behavior.

The controller parameters were tuned systematically to ensure a fair and consistent comparison among the evaluated control strategies. For each controller, the gains were adjusted to achieve a balance between fast convergence, minimal overshoot, and bounded control effort under identical simulation conditions. Additionally, the resulting control torques and joint velocities were monitored to ensure physically realistic behavior. The control inputs remained within bounded limits (approximately within ±[50, 30, 15] Nm for joints 1–3, respectively), and joint velocity constraints were considered to ensure operation within realistic actuator bandwidth limits. The joint velocities were monitored during all simulations and were maintained below 5 rad/s, confirming that no actuator saturation or unrealistic dynamics occurred. The same tuning criteria and performance objectives were applied uniformly to all controllers to ensure a fair and unbiased comparison.

To ensure a fair and meaningful comparison, the role of system dynamics is carefully considered in each control strategy. The PID controller incorporates gravity compensation to improve steady-state accuracy and ensure proper equilibrium behavior. The sliding mode controller is designed based on the full nonlinear dynamic model, including inertia, Coriolis/centrifugal, and gravitational effects, which contributes to its robustness and stability properties. In contrast, the fuzzy logic controller is implemented as a model-free approach, where control actions are generated based on error signals without explicit use of system dynamics. This distinction allows for a comparative evaluation between model-based and model-free control strategies under consistent operating conditions.

### PID control design

PID control is implemented in the joint space and designed independently for each joint. Despite the coupled nature of robotic manipulators, decentralized PID controllers remain widely used in practice due to their simplicity, intuitive tuning, and ease of implementation^8–10^.

The control input for the *i*-th joint is given by

21 $$\:{\boldsymbol{\tau\:}}_{\boldsymbol{i}}\left(\boldsymbol{t}\right)={K}_{p,i}{\boldsymbol{e}}_{\boldsymbol{i}}\left(\boldsymbol{t}\right)+{K}_{d,i}{\dot{\boldsymbol{e}}}_{\boldsymbol{i}}\left(\boldsymbol{t}\right)+{K}_{i,i}{\int\:}_{0}^{t}\:{\boldsymbol{e}}_{\boldsymbol{i}}\left(\boldsymbol{\tau\:}\right)d\tau\:,i=\mathrm{1,2},3$$

To improve steady-state accuracy and reduce gravitational loading effects, a gravity compensation term derived from the dynamic model is added to the PID control law. This modification helps eliminate static positioning errors while preserving the simple structure of the PID controller. However, since the nonlinear coupling and velocity-dependent terms are not explicitly compensated, performance degradation may still occur during fast or highly dynamic motions.

### Fuzzy logic control design

To address the nonlinear, coupled, and configuration-dependent dynamics of the 3-DOF robotic manipulator, a Mamdani-type Fuzzy Logic Controller (FLC) is developed for joint-space trajectory tracking. Unlike model-based controllers, the fuzzy approach does not rely explicitly on the exact dynamic equations. Instead, it approximates nonlinear control behavior using linguistic rules derived from intuitive second-order system response characteristics. The controller is designed independently for each joint to maintain implementation simplicity while ensuring effective tracking performance^11,13,27^.

Let the tracking error for the $\:i$-th joint be defined as:

22 $$\:{\boldsymbol{e}}_{\boldsymbol{i}}\left(\boldsymbol{t}\right)={\boldsymbol{\theta\:}}_{\boldsymbol{d}\boldsymbol{i}}\left(\boldsymbol{t}\right)-{\boldsymbol{\theta\:}}_{\boldsymbol{i}}\left(\boldsymbol{t}\right)$$

and its time derivative:

23 $$\:{\dot{\boldsymbol{e}}}_{\boldsymbol{i}}\left(\boldsymbol{t}\right)={\dot{\boldsymbol{\theta\:}}}_{\boldsymbol{d}\boldsymbol{i}}\left(\boldsymbol{t}\right)-{\dot{\boldsymbol{\theta\:}}}_{\boldsymbol{i}}\left(\boldsymbol{t}\right)$$

The nonlinear mapping is expressed as

24 $$\:{\boldsymbol{\tau\:}}_{\boldsymbol{i}}=\mathcal{F}({\boldsymbol{e}}_{\boldsymbol{i}},{\dot{\boldsymbol{e}}}_{\boldsymbol{i}})$$

**Fig. 2:**
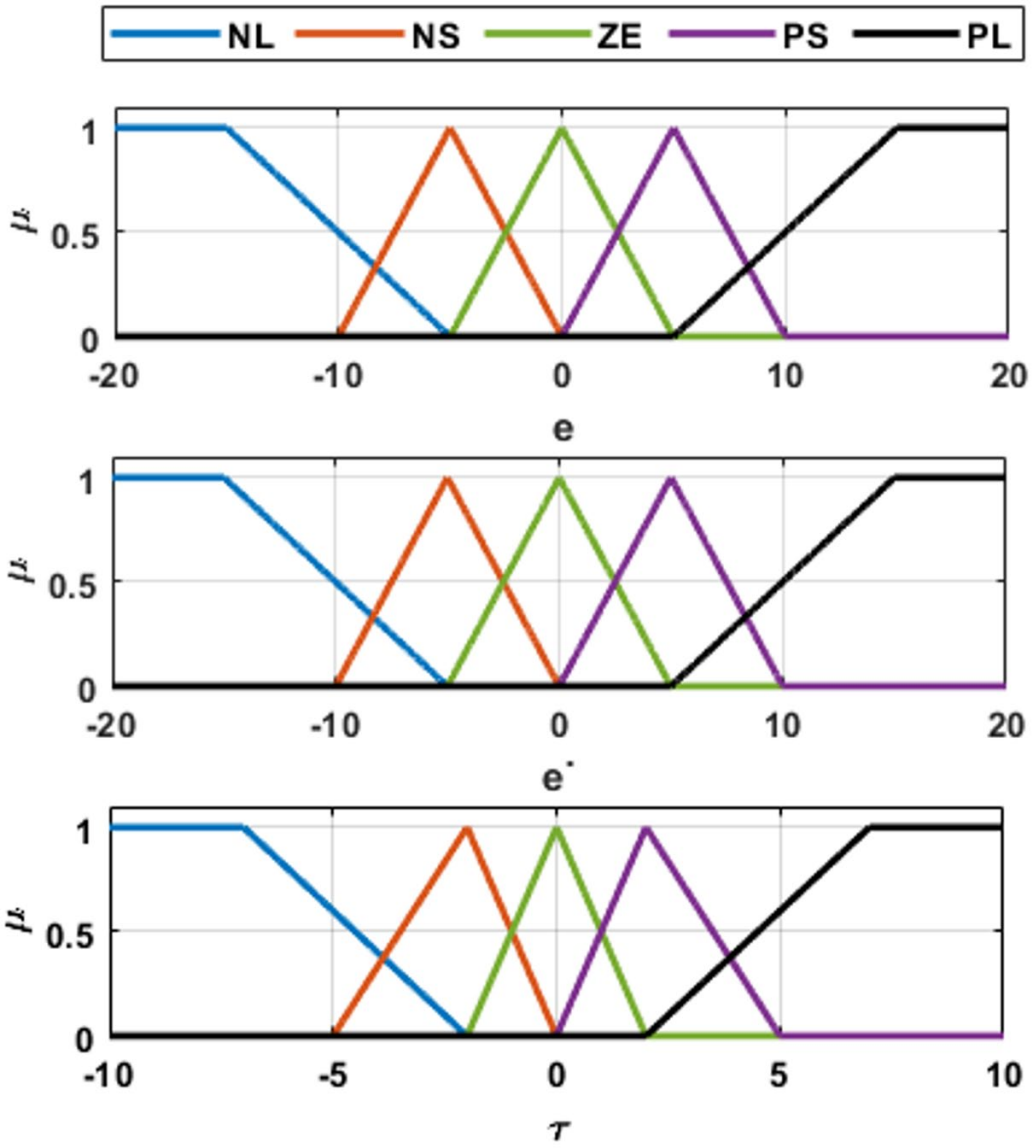
Membership functions of the fuzzy logic controller for the inputs $\:{e}_{1}$, $\:{\dot{e}}_{1}$, and the output torque $\:{\tau\:}_{1}$.

**Fig. 3:**
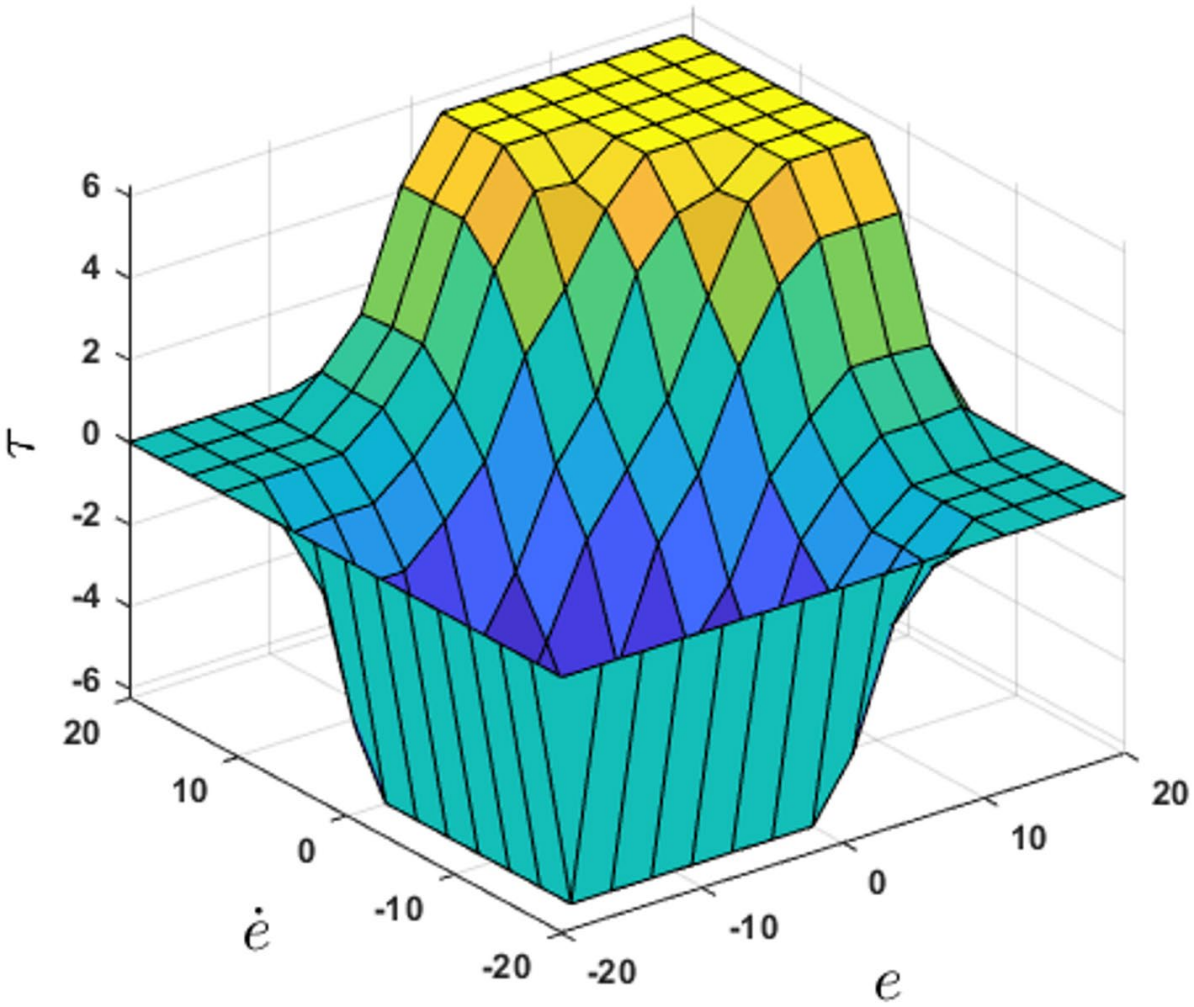
Fuzzy control surface illustrating the nonlinear mapping between tracking error, error derivative, and output torque.

Both inputs were normalized within $\:\left[-\mathrm{20,20}\right]$, while the output torque was bounded in [−10,10]. Five triangular linguistic variables were used for each variable:

{NL, NS, ZE, PS, PL}. The inference mechanism employs Mamdani min–max composition with centroid defuzzification. The membership function distributions are illustrated in Fig. 2, where µ denotes the membership degree, e is the tracking error, ė is the rate of change of the error, and τ represents the control output. The resulting control surface is shown in Fig. 3.

Triangular membership functions are used for to ensure smooth interpolation. The symmetric arrangement of the membership functions ensures balanced control action in positive and negative motion directions.

A 5 × 5 rule matrix (25 rules per joint) is constructed based on intuitive control principles of a damped second-order system as summarized in Table 3.

**Table 3:** Fuzzy rule base used in the FLC design.

e˙∖e	NL	NS	ZE	PS	PL
**NL**	NL	NL	NL	NS	ZE
**NS**	NL	NL	NS	ZE	PS
**ZE**	NL	NS	ZE	PS	PL
**PS**	NS	ZE	PS	PL	PL
**PL**	ZE	PS	PL	PL	PL

The rule design logic is defined as follows:

- Large positive error → large positive corrective torque.
- Large negative error → large negative torque.
- Small error with high derivative → damping action.
- Near-zero error and derivative → minimal torque.

This structure allows the fuzzy controller to emulate a nonlinear PD controller with variable gains depending on the operating region. The following fuzzy inference configuration is used:

- Inference type: Mamdani.
- AND operator: Minimum.
- Aggregation: Maximum.
- Defuzzification method: Centroid (Center of Gravity).

The crisp output torque is computed as^4^:

25 $$\:{\tau\:}_{i}=\frac{{\int\:}_{{\Omega\:}}{\mu\:}_{{\tau\:}_{i}}\left(x\right){\hspace{0.17em}}x{\hspace{0.17em}}dx}{{\int\:}_{{\Omega\:}}{\mu\:}_{{\tau\:}_{i}}\left(x\right){\hspace{0.17em}}dx}$$

To regulate controller aggressiveness and adapt to operating conditions, input and output scaling gains are introduced:

26 $$\:{\tau\:}_{i}\left(t\right)={K}_{\tau\:}{\hspace{0.17em}FLC}\left({K}_{e}{e}_{i}\left(t\right),{K}_{\dot{e}}{\dot{e}}_{i}\left(t\right)\right)$$

Where $\:{K}_{e}$, $\:{K}_{\dot{e}}$, and $\:{K}_{\tau\:}$ denote the error scaling gain, derivative scaling gain, and output torque scaling gain, respectively. These factors adjust the influence of the tracking error, its rate of change, and the overall control amplitude, thereby regulating the responsiveness and stability of the fuzzy controller.

### Sliding mode control

To ensure robust trajectory tracking in the presence of nonlinear coupling, parametric uncertainties, and external disturbances, a Sliding Mode Controller (SMC) is developed for the 3-DOF robotic manipulator. Sliding mode control is particularly attractive for robotic systems due to its strong robustness properties and guaranteed convergence under bounded uncertainties. Unlike PID and fuzzy control, SMC explicitly utilizes the nonlinear dynamic model derived in Sect. 2 and enforces the system trajectories onto a predefined sliding manifold, where the tracking error dynamics exhibit desired stability properties^14–16^. The manipulator dynamics are given by:

27 $$\:\mathbf{M}\left(\boldsymbol{\theta\:}\right){\hspace{0.17em}}\ddot{\boldsymbol{\theta\:}}+\boldsymbol{C}\left(\boldsymbol{\theta\:},\dot{\boldsymbol{\theta\:}}\right)+\mathbf{G}\left(\boldsymbol{\theta\:}\right)\:+\boldsymbol{F}\left(\dot{\boldsymbol{\theta\:}}\right)=\boldsymbol{\tau\:}$$

where $\:\boldsymbol{M}\left(\boldsymbol{\theta\:}\right)$is the inertia matrix, $\:\boldsymbol{C}(\boldsymbol{\theta\:},\dot{\boldsymbol{\theta\:}})$represents the Coriolis and centrifugal terms, $\:\boldsymbol{G}\left(\boldsymbol{\theta\:}\right)$denotes the gravitational torque vector, $\:\boldsymbol{F}\left(\dot{\boldsymbol{\theta\:}}\right)$represents the friction forces, and $\:\boldsymbol{\tau\:}$ is the control torque vector.

To account for modeling inaccuracies and external disturbances, the manipulator dynamics can be expressed in the presence of lumped uncertainty as:

28 $$\:M\left(\theta\:\right)\ddot{\theta\:}+C(\theta\:,\dot{\theta\:})\dot{\theta\:}+G\left(\theta\:\right)+F\left(\dot{\theta\:}\right)=\tau\:+d\left(t\right)$$

where $\:d\left(t\right)$represents the lumped uncertainty term, including unmodeled dynamics, parameter variations, and external disturbances. It is assumed that the uncertainty is bounded such that:

29 $$\:\parallel\:d\left(t\right)\parallel\:\le\:{{\Delta\:}}_{\mathrm{m}\mathrm{a}\mathrm{x}}$$

where $\:{{\Delta\:}}_{\mathrm{m}\mathrm{a}\mathrm{x}}$is a known positive constant.

Let the tracking error be defined as:

30 $$\:\mathbf{e}={\boldsymbol{\theta\:}}_{\boldsymbol{d}}-\boldsymbol{\theta\:},\:\:\dot{\mathbf{e}}={\dot{\boldsymbol{\theta\:}}}_{\boldsymbol{d}}-\dot{\boldsymbol{\theta\:}}$$

The control objective is to ensure:

31 $$\:\mathbf{e}\left(\boldsymbol{t}\right)\to\:0\:\mathrm{as}\text{}t\to\:{\infty\:}$$

A linear sliding surface $\:\mathrm{S}\left(\mathrm{t}\right)\in\:{\mathbb{R}}^{3}$ is defined as:

32 $$\:\mathbf{s}\left(\boldsymbol{t}\right)=\dot{\mathbf{e}}\left(\boldsymbol{t}\right)+\boldsymbol{\Lambda\:}\mathbf{e}\left(\boldsymbol{t}\right)$$

where: $\:\boldsymbol{\Lambda\:}=\mathrm{diag}\left({\lambda\:}_{1},{\lambda\:}_{2},{\lambda\:}_{3}\right)$ is a positive definite diagonal matrix representing the sliding surface slope. When the system reaches the sliding surface ($\:\mathrm{s}=0$), the error dynamics become:

33 $$\:\dot{\mathbf{e}}+\boldsymbol{\Lambda\:}\mathbf{e}=0$$

which guarantees exponential convergence of the tracking error to zero.

The proposed control torque $\:\boldsymbol{\tau\:}$ is composed of an equivalent control term $\:{\boldsymbol{\tau\:}}_{\boldsymbol{e}\boldsymbol{q}}$ and a robust switching term $\:{\boldsymbol{\tau\:}}_{\boldsymbol{s}\boldsymbol{w}}$:

34 $$\:\boldsymbol{\tau\:}={\boldsymbol{\tau\:}}_{\boldsymbol{e}\boldsymbol{q}}+{\boldsymbol{\tau\:}}_{\boldsymbol{s}\boldsymbol{w}}$$

The equivalent control compensates for nominal system dynamics:

35 $$\:{\boldsymbol{\tau\:}}_{\boldsymbol{e}\boldsymbol{q}}=\mathbf{M}\left(\boldsymbol{\theta\:}\right)\left({\ddot{\boldsymbol{\theta\:}}}_{\boldsymbol{d}}+\boldsymbol{\Lambda\:}\dot{\mathbf{e}}\right)+\boldsymbol{C}\left(\boldsymbol{\theta\:},\dot{\boldsymbol{\theta\:}}\right)+\mathbf{G}\left(\boldsymbol{\theta\:}\right)+\:\boldsymbol{F}\left(\dot{\boldsymbol{\theta\:}}\right)\:\:$$

This term ensures ideal tracking under perfect model knowledge.

To handle modeling uncertainties and ensure robustness while eliminating the chattering effect common in classical SMC, a smooth switching law using the hyperbolic tangent function is implemented:

36 $$\:{\boldsymbol{\tau\:}}_{\boldsymbol{s}\boldsymbol{w}}=\mathbf{K}{\hspace{0.17em}tanh}\left(\frac{\boldsymbol{S}}{\boldsymbol{\varphi\:}}\right)$$

where $\:\boldsymbol{K}=\mathrm{d}\mathrm{i}\mathrm{a}\mathrm{g}({k}_{1},{k}_{2},{k}_{3})$is the switching gain matrix and $\:\phi\:$denotes the boundary layer thickness. Unlike the discontinuous $\:\mathrm{s}\mathrm{g}\mathrm{n}\left(\boldsymbol{S}\right)$or the piecewise linear $\:\mathrm{s}\mathrm{a}\mathrm{t}\left(\boldsymbol{S}\right)$functions, the $\:\mathrm{t}\mathrm{a}\mathrm{n}\mathrm{h}(\cdot\:)$function provides a smooth $\:{C}^{{\infty\:}}$transition, which significantly reduces mechanical vibrations and actuator wear. To mitigate the chattering phenomenon commonly associated with classical sliding mode control, a continuous approximation of the switching function is employed using the hyperbolic tangent function instead of the discontinuous sign function. This introduces a boundary layer around the sliding surface, resulting in smoother control action and reduced high-frequency oscillations while maintaining robustness against uncertainties. The complete SMC law becomes:

37 $$\:\boldsymbol{\tau\:}=\boldsymbol{M}\left(\boldsymbol{\theta\:}\right)\left({\ddot{\boldsymbol{\theta\:}}}_{\boldsymbol{d}}+\boldsymbol{\varLambda\:}\dot{\boldsymbol{e}}\right)+\boldsymbol{C}\left(\boldsymbol{\theta\:},\dot{\boldsymbol{\theta\:}}\right)+\boldsymbol{G}\left(\boldsymbol{\theta\:}\right)+\boldsymbol{F}\left(\dot{\boldsymbol{\theta\:}}\right)\:+\boldsymbol{K}{\hspace{0.17em}tanh}\left(\frac{\boldsymbol{S}}{\boldsymbol{\varphi\:}}\right)$$

The selection of the switching gain matrix $\:K$plays a critical role in ensuring robustness of the sliding mode controller. In classical SMC design, $\:K$is chosen to exceed the upper bound of the lumped uncertainties, including modeling errors and external disturbances, in order to satisfy the reaching condition.

This requirement can be expressed as $\:\boldsymbol{K}\ge\:{{\Delta\:}}_{\mathrm{m}\mathrm{a}\mathrm{x}}/\phi\:$, where $\:{{\Delta\:}}_{\mathrm{m}\mathrm{a}\mathrm{x}}$denotes the upper bound of uncertainties and $\:\phi\:$represents the boundary layer thickness. However, in practical applications, exact bounds are often difficult to obtain. The switching gain matrix $\:K$is selected to dominate the effect of lumped uncertainties and external disturbances. Assuming that the disturbance is bounded such that $\:\parallel\:d\left(t\right)\parallel\:\le\:{{\Delta\:}}_{\mathrm{m}\mathrm{a}\mathrm{x}}$, the gain is chosen sufficiently large to satisfy the reaching condition and ensure stability. In practice, the exact value of $\:{{\Delta\:}}_{\mathrm{m}\mathrm{a}\mathrm{x}}$is not precisely known. Therefore, the gain $\:K$is selected based on this condition and then fine-tuned empirically to achieve fast convergence and smooth control action.

To verify the stability of the system, consider the following Lyapunov candidate function:

38 $$\:V=\frac{1}{2}{\mathbf{s}}^{T}\mathbf{s}$$

Taking the time derivative of $\:V$ and substituting the system dynamics and the proposed control law, we obtain:

39 $$\:\dot{V}={\mathbf{s}}^{T}\dot{\mathbf{s}}$$

Assuming $\:K$is chosen sufficiently large to dominate the bounded uncertainties and external disturbances, the derivative satisfies:

40 $$\:\dot{V}\le\:-{\mathbf{s}}^{T}\mathbf{K}{\hspace{0.17em}tanh}\left(\frac{\boldsymbol{S}}{\boldsymbol{\varphi\:}}\right)<\:0\:\mathrm{f}\mathrm{o}\mathrm{r}\:\boldsymbol{S}\ne\:0$$

Considering the bounded uncertainty $\:\parallel\:d\left(t\right)\parallel\:\le\:{{\Delta\:}}_{\mathrm{m}\mathrm{a}\mathrm{x}}$and selecting the gain $\:K$ such that $\:K\ge\:{{\Delta\:}}_{\mathrm{m}\mathrm{a}\mathrm{x}}/\phi\:$, the disturbance effect is dominated by the switching control term. This condition ensures that the reaching condition is satisfied and the system state trajectories are driven toward the sliding surface in finite time, guaranteeing the asymptotic stability of the closed-loop system in the Lyapunov sense.

## Model validation

To further validate the correctness of the implemented model and simulation framework, a second validation test was performed using the periodic joint trajectories reported in the reference study. The desired joint-space trajectories are defined as^26^

41 $$\:{q}_{d1}=\mathrm{s}\mathrm{i}\mathrm{n}\left(0.2\pi\:t\right),{q}_{d2}=\mathrm{s}\mathrm{i}\mathrm{n}(0.2\pi\:t-\frac{\pi\:}{4}),{q}_{d3}=\mathrm{s}\mathrm{i}\mathrm{n}(0.2\pi\:t-\frac{\pi\:}{2})$$

**Fig. 4:**
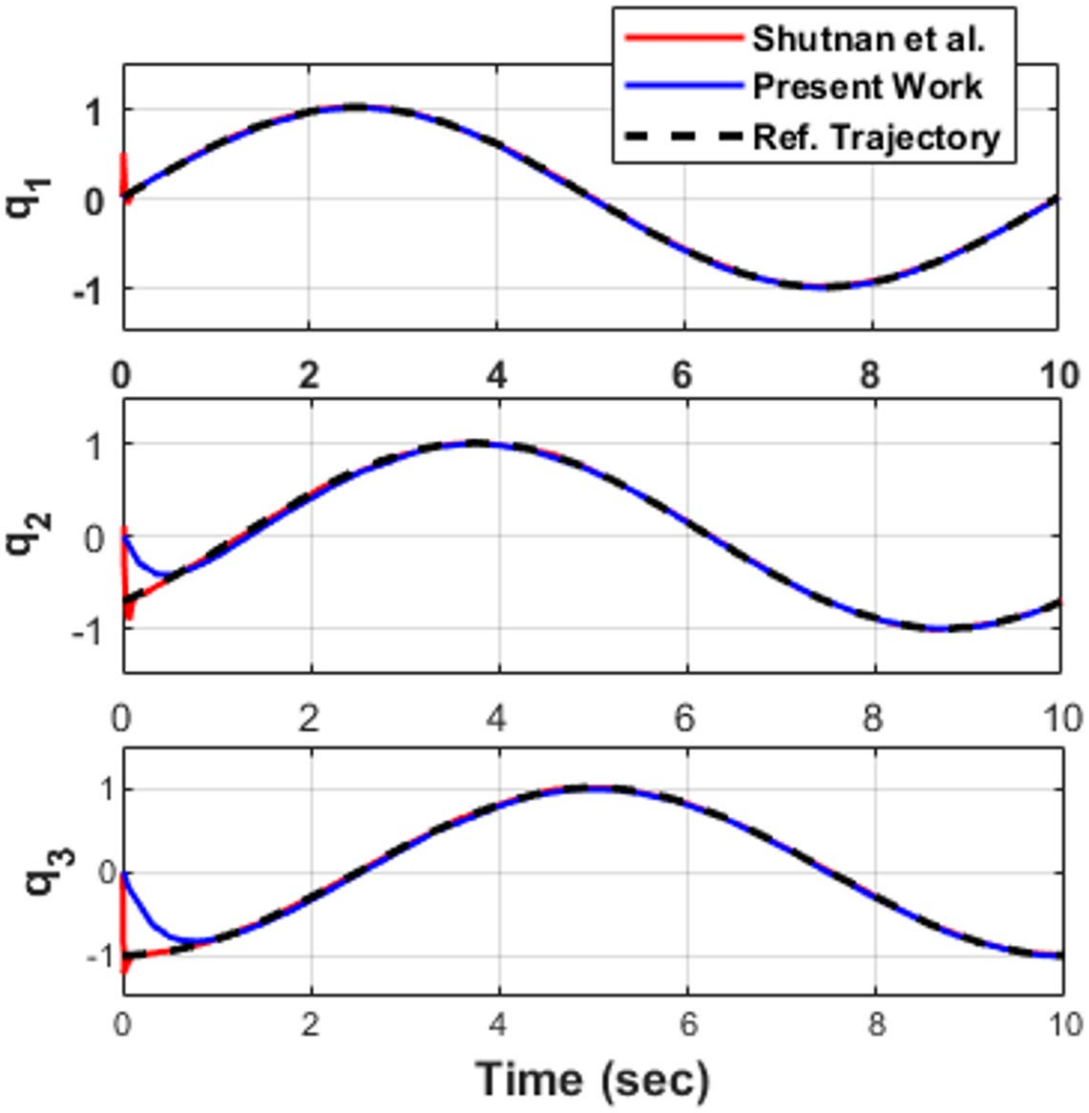
Sinusoidal trajectory validation: published results vs. present work.

These trajectories introduce phase shifts of $\:\pi\:/4$and $\:\pi\:/2$between the joints, creating coordinated multi-axis motion and testing the coupling characteristics of the dynamic model. Figure 4 presents the comparative responses of the reference trajectory, the results reported in Shutnan et al.^26^, and the responses obtained in the present work. The obtained trajectories exhibit identical amplitude (± 1 rad), frequency (0.2π rad/s), and phase relationships across all joints. The present implementation accurately reproduces the inter-joint phase differences, confirming correct handling of dynamic coupling and gravitational interactions. The transient responses show negligible deviation during the initial stage, while steady-state tracking demonstrates very close agreement, with only small deviations between the published results and the present simulation. No noticeable phase distortion or amplitude attenuation is observed, and steady-state error remains negligible within simulation accuracy.

The excellent agreement confirms:

- Correct implementation of the nonlinear inertia and Coriolis matrices.
- Accurate gravity term formulation.
- Proper numerical integration within the Simulink environment.

Therefore, the trajectory validation further establishes the mathematical integrity and numerical reliability of the developed 3-DOF manipulator model, ensuring that subsequent controller performance comparisons are conducted on a verified dynamic platform.

## Results and discussion

This section presents the simulation results and comparative performance evaluation of the PID, Fuzzy Logic, and Sliding Mode controllers applied to the 3-DOF robotic manipulator. The controllers are evaluated under identical simulation conditions in both joint space and Cartesian space. It should be noted that the compared controllers are not structurally equivalent. The sliding mode controller (SMC) utilizes the full nonlinear dynamic model, the PID controller incorporates only gravity compensation, while the fuzzy logic controller (FLC) operates as a model-free approach. Therefore, the comparison reflects practical controller design philosophies rather than strictly equivalent control structures. Performance is analyzed in terms of tracking accuracy, transient response, robustness, and quantitative error metrics. The results provide a systematic comparison that highlights the strengths and limitations of each control strategy under nonlinear dynamic conditions. To ensure a fair and comprehensive comparison, both position and velocity tracking errors are evaluated using identical performance metrics, including RMSE, ITAE, and IAE. The desired velocity trajectory is obtained as the time derivative of the desired position trajectory, ensuring consistency between kinematic references and dynamic performance evaluation.

### Joint-space tracking performance

**Fig. 5:**
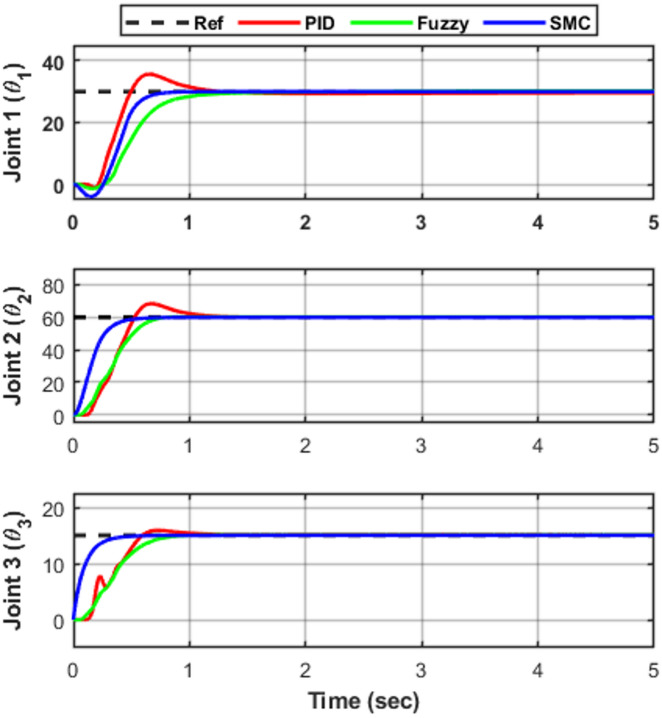
Joint-space step tracking responses of the 3-DOF robotic manipulator under PID, FLC, and SMC.

To evaluate the transient tracking performance of the proposed controllers, step reference inputs were applied to joints $\:{\theta\:}_{1}$, $\:{\theta\:}_{2}$, and $\:{\theta\:}_{3}$. The desired positions were approximately $\:{30}^{\circ\:}$, $\:{60}^{\circ\:}$, and $\:{15}^{\circ\:}$, respectively. Figure 5 illustrates the joint responses under PID, Fuzzy Logic (FLC), and Sliding Mode Control (SMC).

All controllers achieve stable tracking with negligible steady-state error (≈ 0 within simulation precision). However, clear differences appear in the transient characteristics. For Joint 1, the PID controller exhibits a relatively large overshoot of approximately 35% (peak ≈ 40°) and settles in about 1.2 s. The fuzzy logic controller (FLC) significantly improves the damping behavior, reducing the overshoot to around 5%, although the rise time becomes slightly slower (≈ 0.60 s) with a settling time close to 1.0 s. In contrast, the sliding mode controller (SMC) provides a faster response with negligible overshoot and achieves stabilization in approximately 0.8 s. For Joint 2, the PID controller shows an overshoot of about 12% with a settling time near 1.2 s. The FLC again limits the peak deviation to approximately 4%, improving the damping while settling in about 1.0 s. The SMC demonstrates nearly critically damped behavior, reaching the reference with almost zero overshoot and the shortest settling time of approximately 0.7 s. For Joint 3, a similar trend is observed. The PID controller produces noticeable overshoot (≈ 18%) and settles in around 1.1 s. The FLC reduces the overshoot to about 3%, improving stability but with a slightly slower rise time (≈ 0.70 s). The SMC again yields the fastest response, settling within 0.6 s with practically no overshoot.

**Table 4:** Transient performance comparison for joint tracking.

Joint	Controller	Rise time (s)	Overshoot (%)	Settling time (s)
**θ₁**	PID	0.35	35	1.2
	FLC	0.60	5	1.0
	SMC	0.40	≈ 0	0.8
**θ₂**	PID	0.45	12	1.2
	FLC	0.65	4	1.0
	SMC	0.35	≈ 0	0.7
**θ₃**	PID	0.35	18	1.1
	FLC	0.70	3	1.0
	SMC	0.30	≈ 0	0.6

From Table 4, it is evident that the sliding mode controller consistently achieves the smallest overshoot and shortest settling time across all joints. The fuzzy logic controller significantly improves damping compared to PID, reducing overshoot by approximately 70–85% depending on the joint. Although the PID controller remains stable, its fixed-gain structure limits its transient performance under the nonlinear and coupled dynamics of the manipulator system, whereas SMC benefits from explicit nonlinear compensation, resulting in superior transient behavior.

### Joint-space tracking error analysis

**Fig. 6:**
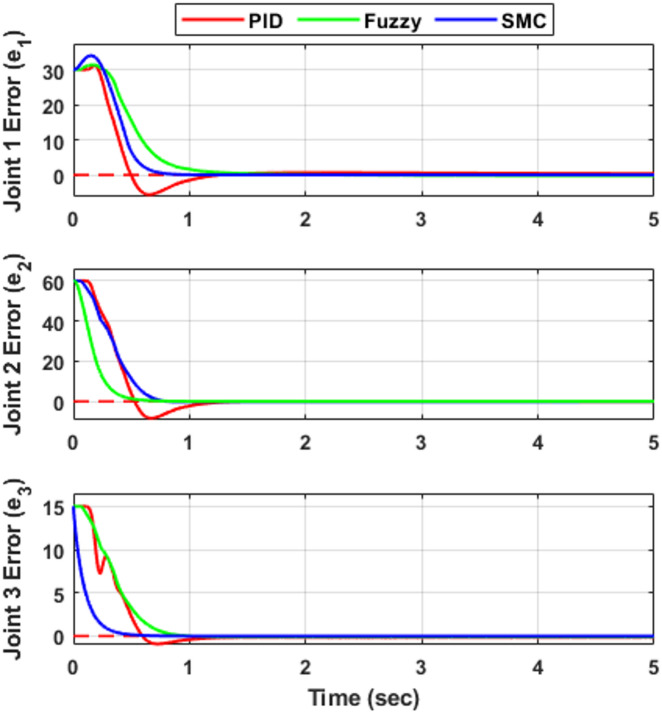
The tracking error responses for joints e₁, e₂, and e₃ under the three controllers.

**Fig. 7:**
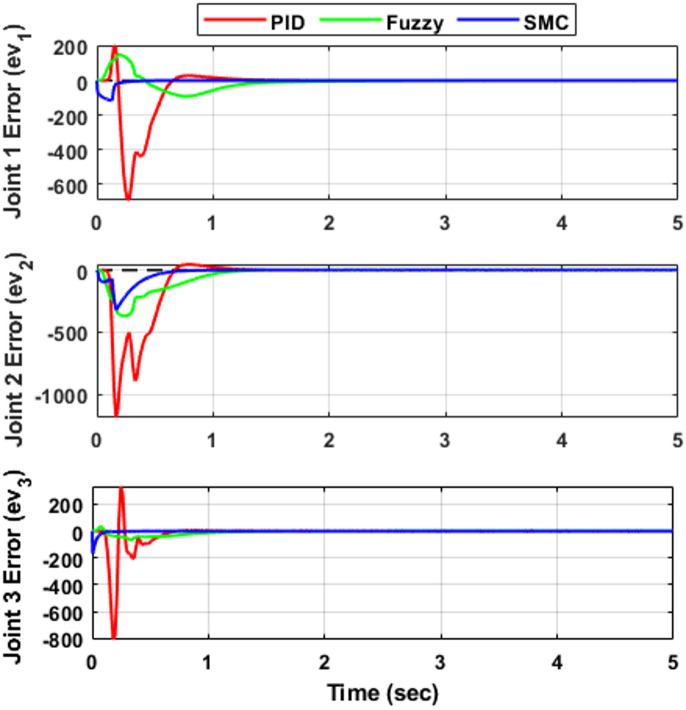
Velocity tracking errors for the three-DOF robotic manipulator using PID, FLC, and SMC.

To further evaluate controller performance, the joint tracking errors corresponding to Fig. 5 are presented in Fig. 6, while the associated velocity tracking errors are shown in Fig. 7. The error responses provide deeper insight into transient behavior, damping characteristics, and convergence speed. Both position and velocity tracking errors are considered to ensure a comprehensive performance assessment. All controllers ultimately achieve negligible steady-state error (≈ 0 within simulation precision) in both position and velocity, confirming stable closed-loop operation. However, noticeable differences appear in transient performance. The PID controller exhibits oscillatory deviations with peak undershoots of approximately − 5°, − 8°, and − 3° for joints $\:{e}_{1}$, $\:{e}_{2}$, and $\:{e}_{3}$, respectively, along with similar oscillatory behavior in the velocity responses shown in Fig. 7. The corresponding settling times are about 1.10 s, 0.95 s, and 0.85 s, indicating relatively slower convergence compared with the other controllers. The fuzzy logic controller (FLC) improves the damping behavior and reduces oscillation amplitude in both position and velocity responses. The settling times are approximately 1.25 s, 0.55 s, and 0.75 s for $\:{e}_{1}$, $\:{e}_{2}$, and $\:{e}_{3}$, respectively, demonstrating faster convergence than PID for joints $\:{e}_{2}$and $\:{e}_{3}$, although the response for $\:{e}_{1}$is slightly slower. The sliding mode controller (SMC) provides the fastest error decay in both position and velocity, forcing the tracking errors to zero within approximately 0.65 s, 0.75 s, and 0.40 s for $\:{e}_{1}$, $\:{e}_{2}$, and $\:{e}_{3}$, respectively, while maintaining minimal oscillations, as evident in Fig. 7. These results highlight the strong robustness and damping capability of SMC, particularly for highly nonlinear and coupled robotic dynamics. Overall, the FLC offers a moderate improvement over classical PID, whereas SMC consistently provides the most rapid and well-damped convergence in both position and velocity tracking.

As summarized in Table 5, SMC consistently achieves the shortest settling time across all joints, followed by FLC, while PID exhibits the slowest convergence.

**Table 5:** Settling time comparison for joint tracking error.

Joint	PID (s)	FLC (s)	SMC (s)
**e₁**	1.10	1.25	0.65
**e₂**	0.95	0.55	0.75
**e₃**	0.85	0.75	0.40

### Infinity trajectory tracking

**Fig. 8:**
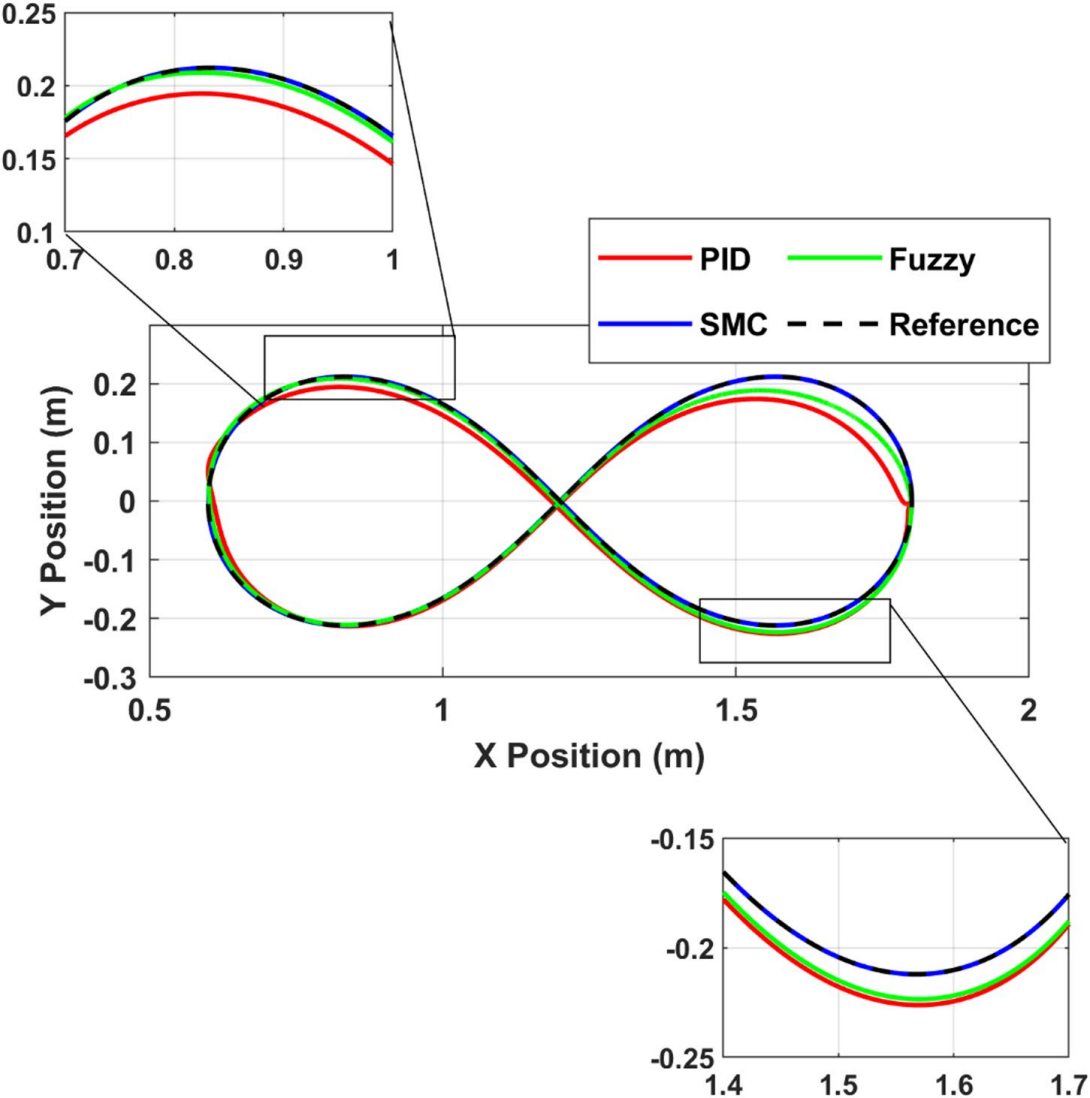
Cartesian tracking performance for the infinity-shaped trajectory under PID, FLC, and SMC.

The Cartesian tracking performance for the infinity-shaped trajectory is illustrated in Fig. 8. The trajectory represents a dynamically demanding path due to continuous curvature variation and direction reversal at the intersection point. All controllers successfully follow the reference path; however, clear deviations are observed at high-curvature regions. The PID controller exhibits visible trajectory lag and deviation, particularly near the turning points of the loops. The fuzzy controller significantly reduces this deviation, producing smoother path convergence. The sliding mode controller demonstrates very close agreement with the reference trajectory, with only small deviations across the entire motion. Zoomed-in views confirm that the largest deviations occur at the extremities of the loops, where acceleration changes are highest.

**Fig. 9:**
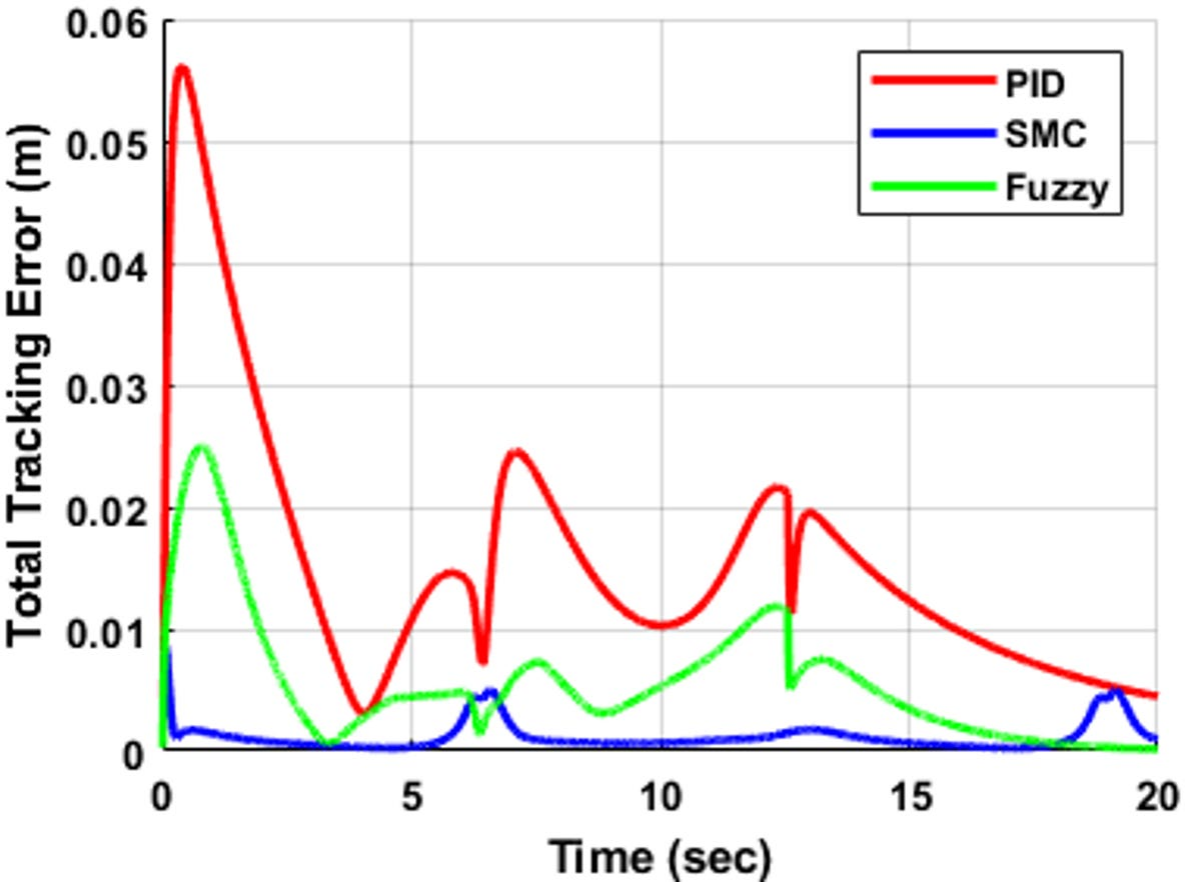
Total Cartesian tracking error for the infinity trajectory.

**Fig. 10:**
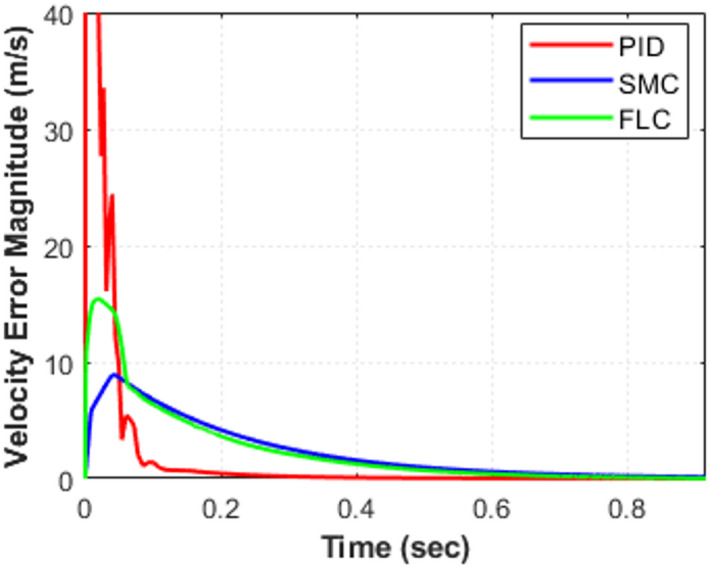
Cartesian velocity tracking errors for PID, FLC, and SMC.

The corresponding total Cartesian tracking error is illustrated in Fig. 9, while the associated Cartesian velocity tracking error is presented in Fig. 10. These results provide a comprehensive evaluation of tracking performance in both position and velocity domains. The PID controller exhibits the largest peak position error, reaching approximately 0.055 m during the initial transient, and continues to display noticeable oscillatory behavior throughout the trajectory, which is also reflected in the velocity responses shown in Fig. 10. In contrast, the fuzzy logic controller (FLC) significantly reduces the peak position error to approximately 0.025 m, representing an improvement of nearly 55% compared to the PID controller, while also providing better damping characteristics in both position and velocity responses. The sliding mode controller (SMC) demonstrates superior performance, maintaining the lowest tracking error, with position error values remaining below 0.005 m for most of the motion and exhibiting rapid attenuation after the transient phase. Similarly, the velocity error in Fig. 10 confirms the fast convergence and minimal oscillatory behavior of SMC. Furthermore, SMC converges to near-negligible steady-state error (≈ 0 within simulation precision) within approximately 4 s, whereas the PID controller continues to suffer from residual oscillations, and the FLC achieves moderate performance with improved stability but slower convergence compared to SMC. The quantitative comparison of peak and steady-state errors for all controllers is summarized in Table 6.

**Table 6:** Infinity trajectory peak tracking error comparison.

Controller	Peak error (m)	Steady-state error (m)
PID	≈ 0.055	≈ 0.005–0.01
FLC	≈ 0.025	≈ 0.003
SMC	≈ 0.008	≈ 0.001

### Circular trajectory tracking

**Fig. 11:**
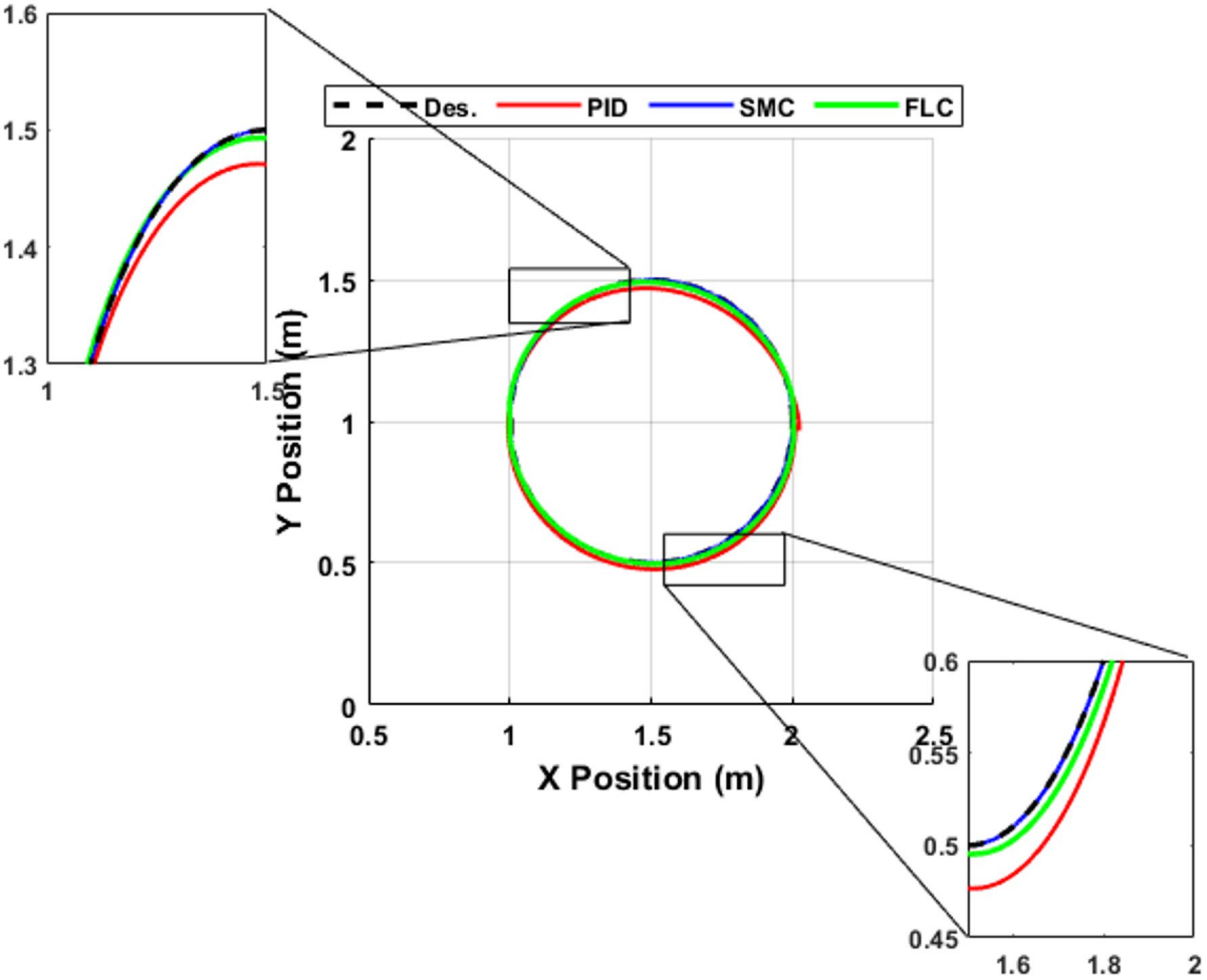
Cartesian tracking performance for the circular trajectory under PID, FLC, and SMC.

The Cartesian tracking results for the circular trajectory are illustrated in Fig. 11. The circular path represents a constant-curvature reference, enabling effective evaluation of the controllers’ ability to maintain accurate tracking under continuous dynamic motion. Although all controllers successfully follow the desired trajectory, clear differences emerge in tracking accuracy, particularly during the initial transient and along high-curvature segments. The PID controller exhibits noticeable deviations from the reference path, especially at the upper and lower portions of the circle. In comparison, the fuzzy logic controller (FLC) reduces these deviations and provides smoother convergence toward the desired trajectory. The sliding mode controller (SMC) demonstrates the highest tracking accuracy, showing very close agreement with the reference path, with only minor deviations throughout the motion.

**Fig. 12:**
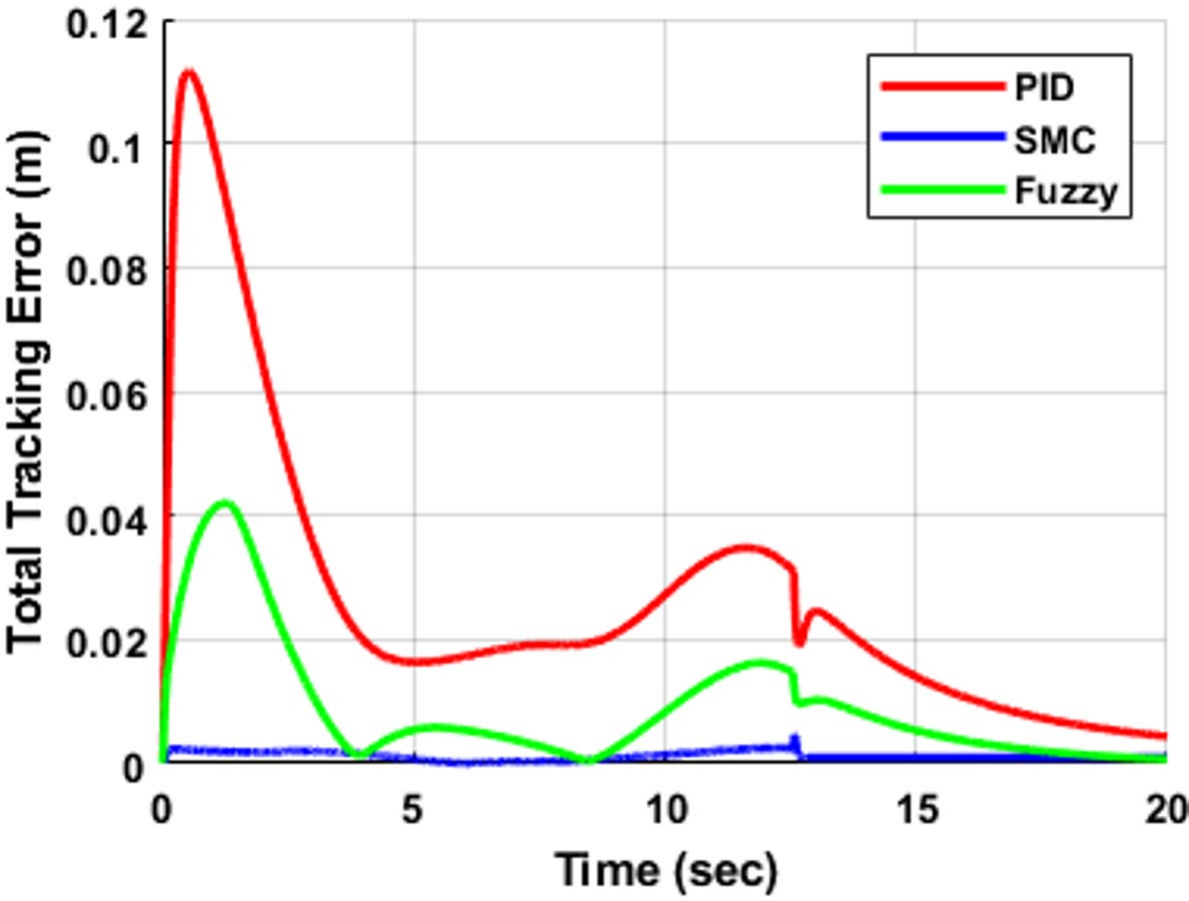
Total Cartesian tracking error for the circular trajectory.

**Fig. 13:**
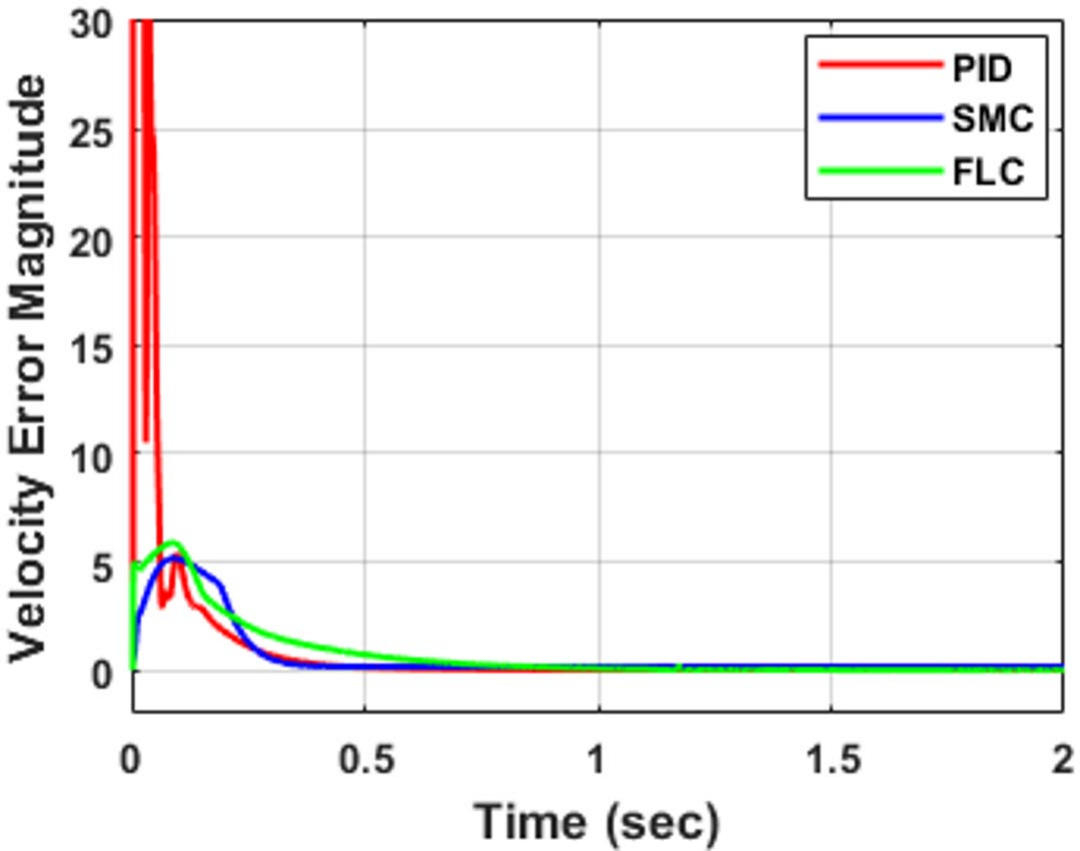
Cartesian velocity tracking errors of the three-DOF robotic manipulator under PID, (FLC), and (SMC).

The corresponding total Cartesian tracking error is presented in Fig. 12, while the associated Cartesian velocity tracking error is shown in Fig. 13. These results provide a comprehensive evaluation of controller performance in both position and velocity domains. The PID controller produces the largest peak position error, reaching approximately 0.11 m during the transient phase, and exhibits noticeable oscillatory behavior, which is also reflected in the velocity responses in Fig. 13. The fuzzy logic controller (FLC) reduces the peak position error to approximately 0.04 m, achieving nearly a 65% improvement compared to PID, while also improving damping characteristics in both position and velocity responses. In contrast, the sliding mode controller (SMC) demonstrates superior performance, maintaining the smallest tracking error, with position error values remaining below 0.005 m for most of the trajectory. Similarly, the velocity error shown in Fig. 13 confirms the rapid convergence and minimal oscillatory behavior of SMC. Furthermore, SMC rapidly converges to near-negligible steady-state error (≈ 0 within simulation precision) within approximately 4 s, while the PID controller continues to exhibit noticeable residual error and oscillatory behavior. The FLC achieves intermediate performance, significantly improving accuracy over PID but remaining less precise than SMC. The quantitative comparison of peak and residual tracking errors is summarized in Table 7.

**Table 7:** Circular trajectory peak tracking error comparison.

Controller	Peak Error (m)	Residual Error (m)
PID	≈ 0.11	≈ 0.005
FLC	≈ 0.04	≈ 0.001
SMC	≈ 0.005	≈ 0.0005

### Quantitative performance comparison

To objectively evaluate the performance of the PID, FLC, and SMC controllers, standard performance indices were computed for both position and velocity tracking in joint space. The Root Mean Square Error (RMSE) quantifies overall tracking accuracy, the Integral of Absolute Error (IAE) reflects cumulative tracking deviation, and the Integral of Time-weighted Absolute Error (ITAE) emphasizes transient performance by penalizing sustained errors over time. These metrics are consistently applied to both position and velocity tracking errors to ensure a unified and fair evaluation framework.

#### RMSE analysis

Tables 8 and 9 summarize the RMSE values for all three joints under the PID, FLC, and SMC controllers for position and velocity tracking, respectively. The results clearly indicate that the SMC achieves the lowest RMSE across all joints, followed by the FLC, while the PID controller exhibits the highest tracking error. This trend is consistent for both position and velocity, confirming the superior accuracy and robustness of the sliding mode controller and the noticeable improvement provided by the fuzzy controller compared to the classical PID approach.

**Table 8:** RMSE comparison for joint position tracking.

Joint	PID	FLC	SMC
θ₁	3.2	1.5	0.6
θ₂	6.5	3.2	1.2
θ₃	1.8	0.9	0.4

**Table 9:** RMSE comparison for joint velocity tracking performance.

Joint	PID	FLC	SMC
$\:\dot{\theta\:}_1$	82.3837	26.3568	12.0097
$\:\dot{{\theta\:}_{2}}$	143.2758	60.9913	34.6120
$\:\dot{{\theta\:}_{3}}$	62.9950	11.3279	7.3009

For position tracking, the average RMSE values are approximately 3.83° for PID, 1.87° for FLC, and 0.73° for SMC. Compared to PID, the fuzzy controller reduces the average RMSE by about 51%, while SMC achieves an overall reduction of nearly 81%. The most significant improvement is observed in Joint 2, where the RMSE decreases from 6.5° under PID control to 1.2° with SMC, demonstrating its superior capability in handling nonlinear coupling and dynamic interactions. Similarly, the velocity RMSE results show a consistent trend, where SMC maintains the lowest error values, followed by FLC and PID. The reduction in velocity tracking error further confirms the fast convergence and strong damping characteristics of the SMC, while the FLC provides moderate improvement over PID with reduced oscillations.

#### ITAE analysis

Tables 10 and 11 present the ITAE values for all joints under the three control strategies for position and velocity tracking, respectively. The results indicate that SMC consistently achieves the lowest ITAE, demonstrating faster error attenuation and improved transient performance, while FLC significantly reduces ITAE compared to PID. This consistent trend across both position and velocity responses confirms the superior dynamic performance of SMC and the improved behavior of the fuzzy controller.

**Table 10:** ITAE comparison for joint position tracking.

Joint	PID	FLC	SMC
θ₁	8.5	4.2	1.6
θ₂	15.2	7.8	3.1
θ₃	4.1	2.3	1.0

**Table 11:** ITAE comparison for joint velocity tracking.

Joint	PID	FLC	SMC
$\:\dot{{\theta\:}_{1}}$	12.4580	3.1240	0.8560
$\:\dot{{\theta\:}_{2}}$	22.8950	7.4520	1.9420
$\:\dot{{\theta\:}_{3}}$	8.1240	1.8450	0.4210

For position tracking, the PID controller exhibits the highest ITAE values, reflecting slower convergence and sustained tracking deviations. The average ITAE values are approximately 9.27 for PID, 4.77 for FLC, and 1.90 for SMC. Compared to PID, sliding mode control achieves nearly a 79% reduction in ITAE, demonstrating significantly faster error attenuation and enhanced damping characteristics. The fuzzy controller also provides substantial improvement, reducing ITAE by approximately 48% relative to PID. Similarly, the velocity ITAE results show a consistent pattern, where SMC achieves the lowest values, followed by FLC and PID. This further confirms the rapid convergence and strong damping capability of SMC, while FLC offers moderate improvement with reduced transient oscillations compared to the classical PID controller.

#### IAE analysis

Tables 12 and 13 summarize the IAE values for all joints under the PID, fuzzy logic (FLC), and sliding mode control (SMC) strategies for position and velocity tracking, respectively. The results show a consistent trend with the RMSE and ITAE metrics, where SMC achieves the lowest error values across all joints, followed by FLC, while PID exhibits the highest accumulated error. This behavior is observed in both position and velocity responses, confirming the effectiveness of SMC in minimizing cumulative tracking errors. For position tracking, SMC significantly reduces the total error compared to PID, while FLC provides moderate improvement. A similar trend is observed in velocity tracking, where SMC maintains the lowest IAE values, indicating faster error attenuation and improved dynamic response, whereas FLC reduces the error relative to PID but remains less effective than SMC. Overall, these results confirm the superior tracking accuracy and robustness of the sliding mode controller, as well as the improved performance of the fuzzy logic controller compared to the classical PID approach.

**Table 12:** IAE comparison for joint position tracking.

Joint	PID	FLC	SMC
θ₁	12.196	10.306	6.817
θ₂	10.007	9.6136	6.5367
θ₃	3.2734	2.9935	1.0708

**Table 13:** IAE comparison for joint velocity tracking.

Joint	PID	FLC	SMC
$\:\dot{{\theta\:}_{1}}$	44.711	43.690	29.998
$\:\dot{{\theta\:}_{2}}$	76.727	60.778	60.009
$\:\dot{{\theta\:}_{3}}$	21.009	16.167	15.030

#### Statistical performance analysis

**Fig. 14:**
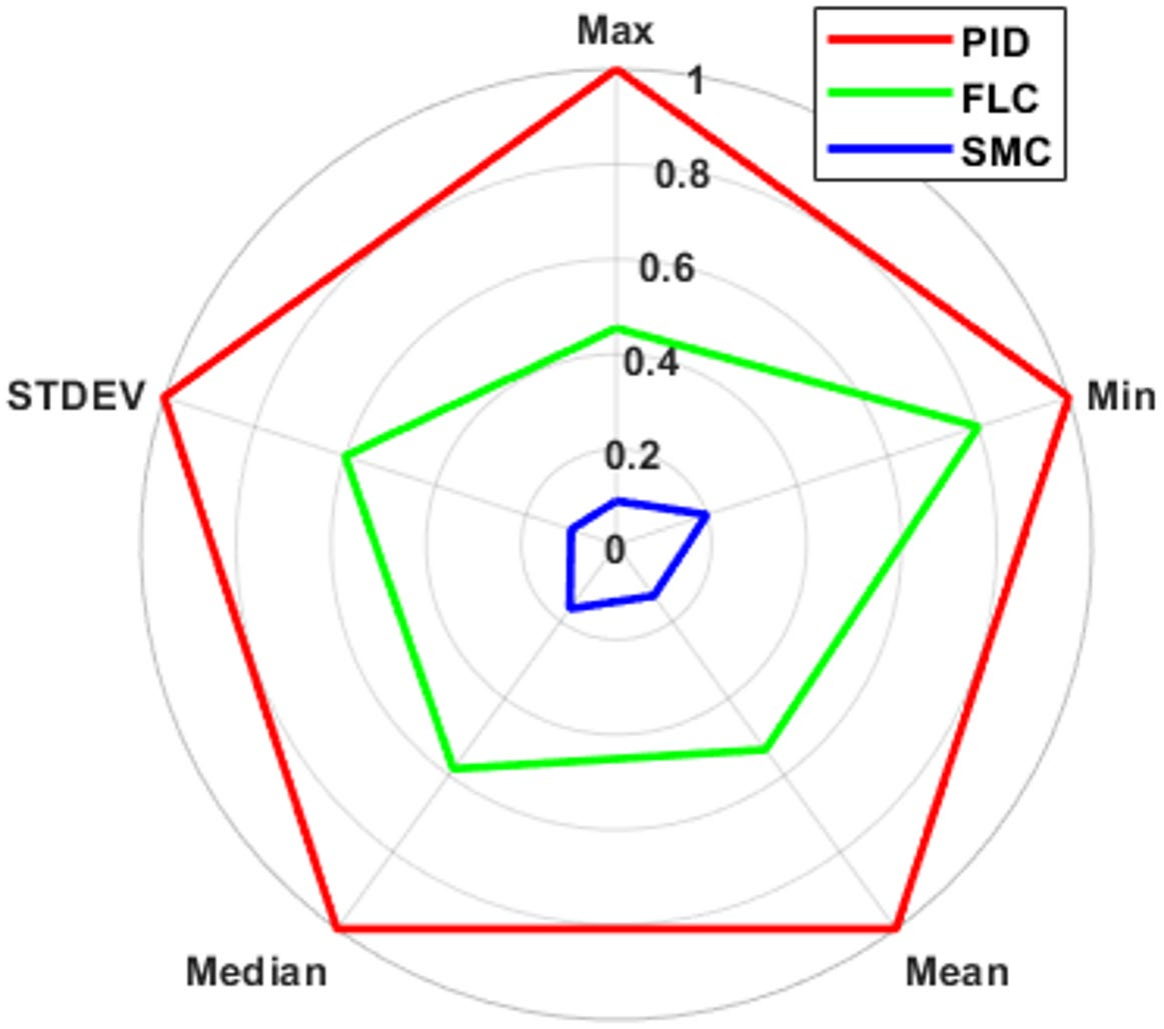
Radar representation of normalized statistical error metrics for PID, FLC, and SMC controllers.

To further quantify controller performance, five statistical indicators were evaluated from the Cartesian tracking error: maximum error (Max), minimum error (Min), mean error, median error, and standard deviation (STDEV). These metrics provide a comprehensive description of peak deviation, central tendency, and error dispersion. As illustrated in Fig. 14, the sliding mode controller occupies the smallest normalized region across all statistical metrics, indicating superior tracking accuracy and reduced variability. In contrast, PID spans the largest area, reflecting higher peak error and greater dispersion. The fuzzy controller exhibits intermediate performance, significantly improving over PID while remaining slightly less precise than SMC. Specifically, SMC achieves the lowest maximum error and standard deviation, demonstrating enhanced robustness during transient phases and improved consistency throughout the trajectory. The reduced mean and median values further confirm its superior steady-state accuracy. Overall, the radar analysis visually reinforces the quantitative results obtained from RMSE and ITAE metrics and confirms the performance ranking: $\:\mathrm{SMC}>\mathrm{FLC}>\mathrm{PID}$. It should be noted that the performance of the evaluated controllers is influenced by the selection of tuning parameters. Although a consistent tuning methodology was applied to ensure fairness, different tuning strategies may lead to variations in performance. Therefore, the presented results reflect the relative behavior of the controllers under the selected tuning conditions.

## Conclusion

This study presented a comprehensive comparative evaluation of PID, Fuzzy Logic (FLC), and Sliding Mode Control (SMC) strategies for a nonlinear 3-DOF robotic manipulator. The controllers were assessed under identical simulation conditions using joint-space tracking, Cartesian infinity and circular trajectories, and quantitative statistical performance indices. The main outcomes of the study can be summarized as:

- All controllers achieved stable joint-space tracking with negligible steady-state error (approximately zero within simulation precision).
- The PID controller exhibited the largest overshoot (up to approximately 20%) and the longest settling time (≈ 1–1.2 s), indicating limited capability in handling nonlinear coupling and dynamic interactions.
- The fuzzy logic controller significantly reduced overshoot and oscillatory behavior, achieving approximately 50–75% reduction in overshoot compared with PID while improving damping characteristics.
- The sliding mode controller achieved the fastest convergence (≈ 0.6–0.7 s) with negligible overshoot and superior transient response across all joints.
- In Cartesian trajectory tracking, SMC maintained the smallest peak error (≈ 0.005 m), while PID exhibited peak deviations reaching ≈ 0.11 m during circular motion.
- RMSE analysis showed that SMC reduced the average tracking error from 3.83° (PID) to 0.73°, corresponding to nearly 81% improvement, while FLC reduced the error to 1.87° (≈ 51% improvement).
- ITAE results confirmed significantly faster error attenuation under SMC, reducing the average ITAE from 9.27 (PID) to 1.90, representing approximately 79% improvement, while FLC achieved ≈ 48% reduction.
- Statistical indicators, including maximum error, mean, median, and standard deviation, consistently validated the overall performance ranking $\:\mathrm{S}\mathrm{M}\mathrm{C}>\mathrm{F}\mathrm{L}\mathrm{C}>\mathrm{P}\mathrm{I}\mathrm{D}$.

Overall, the results demonstrate that robust nonlinear control provides substantial improvements in tracking accuracy, convergence speed, and consistency for planar 3-DOF manipulators operating under dynamic trajectories. This study is limited to simulation-based validation; therefore, the reported results may not fully capture real-world implementation effects such as sensor noise, actuator dynamics, and hardware constraints.

## Data Availability

The data supporting the findings of this study are included in this paper.
